# MPCID, A new high-resolution multi-precipitation concentration indicators dataset for mainland China

**DOI:** 10.1038/s41597-025-06515-2

**Published:** 2026-01-06

**Authors:** Dongyang Zhang, Xuemei Li, Lanhai Li, Yuanlong Tang, Guigang Wang, Huane Duan, Chuanming Yang, Xiaoxiao Jiang

**Affiliations:** 1 Faculty of Geomatics, Lanzhou Jiaotong University, Lanzhou, 730070 China; 2 National-Local Joint Engineering Research Center of Technologies and Applications for National Geographic State Monitoring, Lanzhou, 730070 China; 3 Key Laboratory of Science and Technology in Surveying & Mapping, Gansu Province, Lanzhou, 730070 China; 4 Zhejiang University of Technology, Hangzhou, 310014 China

**Keywords:** Hydrology, Climate-change policy, Natural hazards, Hydrology

## Abstract

Global climate change is intensifying the hydrological cycle, manifested through an increased frequency of extreme precipitation events that pose substantial threats to water security and ecosystem resilience. Precipitation concentration indicators are critical for diagnosing these changes, yet their application has been constrained by data limitations: a reliance on fragmented station observations and a critical disconnect between historical benchmarks and future projections. To bridge this gap, we present the multi-precipitation concentration indicators dataset (MPCID) for mainland China, a spatiotemporally continuous resource spanning 1961–2100. MPCID integrates historical *in-situ* and gridded observations (1961–2022) with high-resolution (0.25°), statistically downscaled CMIP6 projections across four SSP scenarios (2015–2100). The dataset incorporates four key indicators: the precipitation concentration degree (PCD), precipitation concentration period (PCP), daily precipitation concentration index (DPCI), and monthly precipitation concentration index (MPCI). Rigorous validation against station data established PCD as the most reliable indicator, characterized by minimal errors, near-optimal correlation, and negligible bias across both historical and future scenarios. While DPCI exhibited moderate error control, its limited daily-scale correlation points to inherent stochasticity in short-term precipitation. MPCI demonstrated reduced sensitivity to extreme precipitation events, whereas PCP showed systematic limitations in temporal phase alignment despite retaining pattern recognition capability. By integrating historical fidelity with future scenarios, MPCID overcomes prior data fragmentation and establishes an indispensable foundation for investigating precipitation dynamics, assessing climate impacts on hydrology and agriculture, and informing adaptive management strategies.

## Background & Summary

Precipitation, as the principal flux in terrestrial hydrological cycles, functions as an essential climate variable that plays a critical role in regulating global water and energy balances.

Multidimensional temporal precipitation properties, encompassing intensity, frequency, and duration, serve as emerging diagnostic tools for evaluating responses of the climate system to anthropogenic and natural forcings. Global atmospheric warming has been demonstrated to perturb hydrological energy budgets, thereby accelerating moisture recycling rates and amplifying the severity of compound extreme events^1,2^. Benchmarking across Chinese river basins has revealed that extreme precipitation events have exhibited positive responses to anthropogenic warming at rates exceeding 20% / K in southeastern regions, while arid northwestern basins have shown amplified flood risks associated with elevated winter snowfall regimes^3^. Such intensifying hydrological extremes are fundamentally reconfiguring aquatic biogeochemical regimes and watershed resilience capacities^4,5^.

While conventional extreme precipitation indicators (Rx5day for 5-day maximum precipitation, R95p for precipitation exceeding the 95th percentile) effectively characterize the magnitude of extreme events^1^, they primarily focus on “intensity or total amount” rather than the “temporal clustering” of precipitation—a key factor driving drought-flood alternations (prolonged drought followed by flash floods) that severely impact watershed resilience^2^.

Recent advances have highlighted that asymmetrical precipitation distributions critically modulate the dynamics of drought-flood alternations, thereby demanding systematic characterization of precipitation concentration patterns to inform adaptive watershed management strategies.

Indicators such as the monthly precipitation concentration index (MPCI) and daily precipitation concentration index (DPCI) have been developed to characterize the scaling properties of precipitation. The MPCI, initially developed using entropy-based analysis, categorizes spatiotemporal precipitation seasonality into distinct regimes governed by exponential scaling laws^6,7^. This analytical framework has been applied globally to characterize precipitation scaling patterns across diverse climate gradients, such as monsoon-dominated hydrological systems^8,9^. Building on Lorenz curve asymmetry quantification, the DPCI metric has been validated through global benchmarking efforts that capture Mediterranean dryland extremes, Andean rainfall gradients, and East Asian monsoon pulsations^8,10–13^.

To achieve temporal disaggregation of precipitation extremes, precipitation concentration indicators act as essential tools for theoretical decomposition of hydrological processes. Among these, the precipitation concentration degree (PCD) and precipitation concentration period (PCP) enable systematic spatiotemporal analysis of wet season onsets^14^. These indicators characterize convective precipitation clustering and diurnal scaling behaviors, thereby enhancing operational predictability for flash flood early warning systems. Observational records have demonstrated that spatiotemporal decoupling of PCD reduces soil moisture drought persistence, whereas phase advancement in PCP modulates cyclonic rainfall intensification rates^15,16^. Concentrated summer precipitation reductions have amplified drought vulnerabilities in northern basins, while extreme precipitation clustering in coastal regions has accelerated flood recurrence intervals beyond historical norms^3^.

Notably, precipitation concentration indicators address the limitations of conventional extreme indicators by quantifying the spatiotemporal unevenness of precipitation. For example, two years with identical Rx5day values may differ drastically in drought-flood risk: one with rainfall evenly distributed across the year and another with 80% of precipitation concentrated in 3 days, a distinction that Rx5day fails to capture but that PCD / MPCI can explicitly characterize^6,8^. This unique ability to resolve “temporal distribution patterns” makes concentration indicators indispensable for guiding adaptive water management and agricultural risk assessment.

Precipitation concentration research currently faces two persistent methodological constraints. First, current analyses remain fragmented between event-scale diagnostics and climate-scale projections due to the lack of vertically consistent data architectures spanning sub-daily to decadal resolutions. Although advanced indicators have been developed, analytical frameworks remain restricted by traditional ground-based observational paradigms^17–19^. Discrete station data introduce inherent spatial sampling biases, especially across complex topographic gradients such as the Tibetan Plateau, where sparse monitoring networks are insufficient to resolve orographic precipitation enhancement mechanisms^20^. Second, existing precipitation products exhibit incoherence between historical and future datasets, as coarse-resolution CMIP6 outputs are inadequately coupled with statistical downscaling frameworks, thereby introducing uncertainties in extreme event attribution under Shared Socioeconomic Pathways (SSPs)^21^.

To address these limitations, an integrated multi-precipitation concentration indicators dataset (MPCID) has been developed for China, harmonizing *in situ* measurements and scenario-coupled climate projections. This framework bridges the critical gap between historical benchmarks and future climate trajectories through spatiotemporally consistent data representation. The workflow introduces four methodological innovations. First, it integrates 651 quality-controlled stations with 0.25° CN05.1 grid observations to address spatial discontinuities in traditional station-centric analyses. Second, it ensures spatiotemporal extensibility by projecting trends up to 2100 under four SSP scenarios and statistically downscaling CMIP6 outputs to 0.25° resolution, thereby providing high-precision references for future precipitation trends and enabling seamless continuity between historical and future datasets. Third, it generates grid data by integrating latitude / longitude coordinates with k-dimensional tree (KD Tree) and inverse distance weighting (IDW) interpolation, using station observations as benchmarks, followed by systematic accuracy evaluations of the gridded data. It should be noted that this interpolation approach, along with the bilinear resampling applied to global climate models (GCMs) outputs, may smooth local precipitation gradients, particularly in regions with complex topography or sparse station coverage. Fourth, it enables dynamic risk characterization by systematically quantifying spatiotemporal precipitation extremes across China, providing critical insights for climate-resilient policymaking. Openly accessible via figshare (10.6084/m9.figshare.28656086)^22^, MPCID not only overcomes the limitations of prior fragmented data resources but also establishes a replicable framework for global precipitation concentration research^23^.

## Methods

### Data sources

**Fig. 1:**
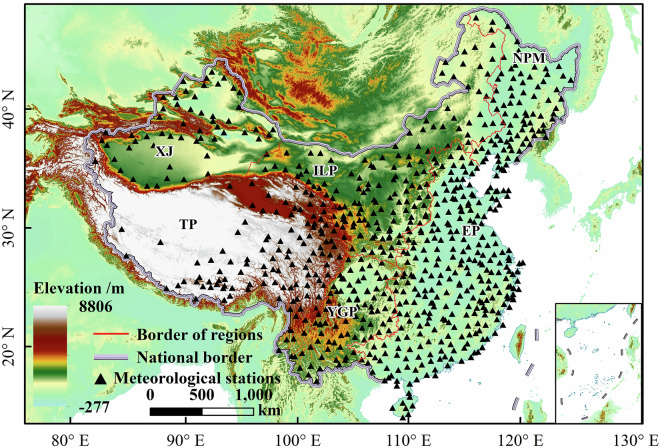
Map of the study area showing the station distributions. Elevation data were derived from SRTM, with map sources from the National Geomatics Center of China (Approval No. GS (2024) 0650; https://cloudcenter.tianditu.gov.cn/dataSource).

Daily precipitation data spanning 1961 to 2020 were sourced from the China ground climate dataset (V3.0; China Meteorological Administration)^24^. Maintained by the National Climate Center of the China Meteorological Administration (CMA), this dataset is publicly accessible at https://data.cma.cn. Due to rigorous quality control implemented prior to release, it was widely used in precipitation concentration studies. To ensure data completeness and continuity, station records were first tested for homogeneity. Data points with a precipitation value of 32766 (denoting missing data in the source dataset) were marked as missing and excluded from further analysis. Stations were excluded if missing daily precipitation data exceeded 5% of the total records or if continuous daily records were absent for more than one year within the study period. After screening, 651 stations were selected as ground truth data for this study (Fig. 1). These stations were distributed across six geographic regions as follows: Tibetan Plateau (TP, n = 52), Xinjiang (XJ, n = 40), Inner Mongolia-Loess Plateau (ILP, n = 117), Northeastern Plain and Mountainous Region (NPM, n = 82), Eastern Plains (EP, n = 277), and Yunnan-Guizhou Plateau (YGP, n = 83). Missing data were addressed using two estimation methods. For gaps of no more than two consecutive days, the average precipitation from adjacent days served as a substitute. For gaps longer than two days, missing values were estimated via simple linear regression using data from neighboring stations with a correlation coefficient exceeding 0.95 (R² > 0.95)^12^. Meteorological stations in the Macao Special Administrative Region and Taiwan Region of China were excluded from this study.

The spatial distribution of meteorological stations in the study area exhibits regional heterogeneity, with sparse station coverage in high-altitude zones (particularly in TP with only 52 stations) and dense observational networks in low-lying plains (notably in EP with 277 stations). The spatial unevenness and sampling limitations of station data hinder the derivation of comprehensive spatial characteristics for precipitation concentration indicators. To project future climate conditions, we downscaled CMIP6 outputs under SSPs. Thus, high-resolution gridded observations were needed to fit and correct the model output. To address these issues, this study utilized the CN05.1 grid dataset (Climate Change Research Center, Chinese Academy of Sciences, https://ccrc.iap.ac.cn/resource/detail?id=228)^25^. Spanning from 1961 to 2022, the dataset reflects climate change trends over the past 60 years. Researchers generated it by combining data from 2,400 stations with grid climatology^26,27^. The dataset has a horizontal resolution of 0.25° × 0.25°, providing detailed climate information for China. In this study, the daily precipitation data from CN05.1 were used primarily as a high-resolution gridded background to supplement the spatial coverage and to fit and correct the GCMs outputs. However, all key validations and conclusions regarding the accuracy of precipitation concentration indicators rely on direct comparisons with the ground-station observations, which serve as the primary benchmark.

Table 1 lists the basic details of the 24 CMIP6 GCMs used in this study (CMIP6 Community, https://esgf-ui.ceda.ac.uk/cog/search/cmip6-ceda/)^28^. Four shared socioeconomic pathways scenarios were considered: SSP1-2.6, SSP2-4.5, SSP3-7.0, and SSP5-8.5. These scenarios represent different socio-economic development pathways and associated levels of radiative forcing^29^. Notably, some GCMs only provide output at monthly resolution. For these models, monthly data were resampled to daily resolution using linear interpolation for consistency in the multi-model ensemble (MME) construction. However, this resampled daily data from monthly-output GCMs was solely used in the historical validation and MME averaging process.

**Table 1:** CMIP6 models used in this study.

No.	Model name	Institution/Country (or region)	Spatial resolution (longitude × latitude)
1	ACCESS-CM2	CSIRO/Australia	1.875° × 1.250°
2	ACCESS-ESM1-5	CSIRO/Australia	1.875° × 1.250°
3	AWI-CM-1-1-MR	AWI/Germany	0.974° × 0.974°
4	AWI-ESM-1-1-LR	AWI/Germany	1.875° × 1.875°
5	BCC-CSM2-MR	BCC/China	1.125° × 1.125°
6	CanESM5	CCCma/Canada	2.813° × 2.813°
7	CAS-ESM2-0	CAS/China	1.406° × 1.406°
8	CESM2-WACCM	NCAR/America	2.500° × 1.875°
9	CMCC-CM2-SR5	CMCC/Italy	1.250° × 0.974°
10	CMCC-ESM2	CMCC/Italy	1.250° × 0.974°
11	FGOALS-g3	CAS/China	2.000° × 2.250°
12	GFDL-ESM4	GFDL/America	1.250° × 1.000°
13	EC-Earth3	EC-Earth-Consortium/EU	0.703° × 0.703°
14	EC-Earth3-Veg	EC-Earth-Consortium/EU	0.703° × 0.703°
15	INM-CM4-8	INM/Russia	2.000° × 1.500°
16	INM-CM5-0	INM/Russia	2.000° × 1.500°
17	IPSL-CM6A-LR	IPSL/France	2.500° × 1.259°
18	KACE-1-0-G	NIMS/Korea	1.875° × 1.250°
19	MIROC6	MIROC/Japan	1.406° × 1.406°
20	MPI-ESM1-2-HR	MPI-M/Germany	0.974° × 0.974°
21	NESM3	NUIST/China	1.875° × 1.875°
22	NorESM2-MM	NCC/Norway	1.250° × 0.974°
23	SAM0-UNICON	SUN/Korea	1.250° × 0.974°
24	TaiESM1	RCEC/China	1.250° × 0.974°

Note: CSIRO, Commonwealth Scientific and Industrial Research Organization; AWI, Alfred Wegener Institute; BCC, Beijing Climate Center; CCCma, Canadian Centre for Climate Modelling and Analysis; CAS, Chinese Academy of Sciences; NCAR, National Center for Atmospheric Research; CMCC, Euro-Mediterranean Centre on Climate Change; EC-Earth-Consortium, European Consortium for Earth System Modeling; EU, European Union; GFDL, Geophysical Fluid Dynamics Laboratory; INM, Institute for Numerical Mathematics; IPSL, Institut Pierre-Simon Laplace; NIMS, National Institute of Meteorological Sciences; MIROC, Model for Interdisciplinary Research on Climate; MPI-M, Max Planck Institute for Meteorology; NUIST, Nanjing University of Information Science and Technology; NCC, National Climate Centre; SUN, Seoul National University; RCEC, Research Center for Environmental Changes. The following models in the GCMs dataset used in this study provide monthly resolution data: AWI-CM-1-1-MR (No. 3), AWI-ESM-1-1-LR (No. 4), CAS-ESM2-0 (No. 7), GFDL-ESM4 (No. 12), NESM3 (No. 21), and SAM0-UNICON (No. 23).

All third-party datasets used in this study are openly available, and their reuse complies with the terms and conditions of the respective data providers (CMIP6 data follows the ESGF data sharing policy; CN05.1 data adheres to the access guidelines of the Climate Change Research Center, Chinese Academy of Sciences).

### Data preprocessing and validation framework

**Fig. 2:**
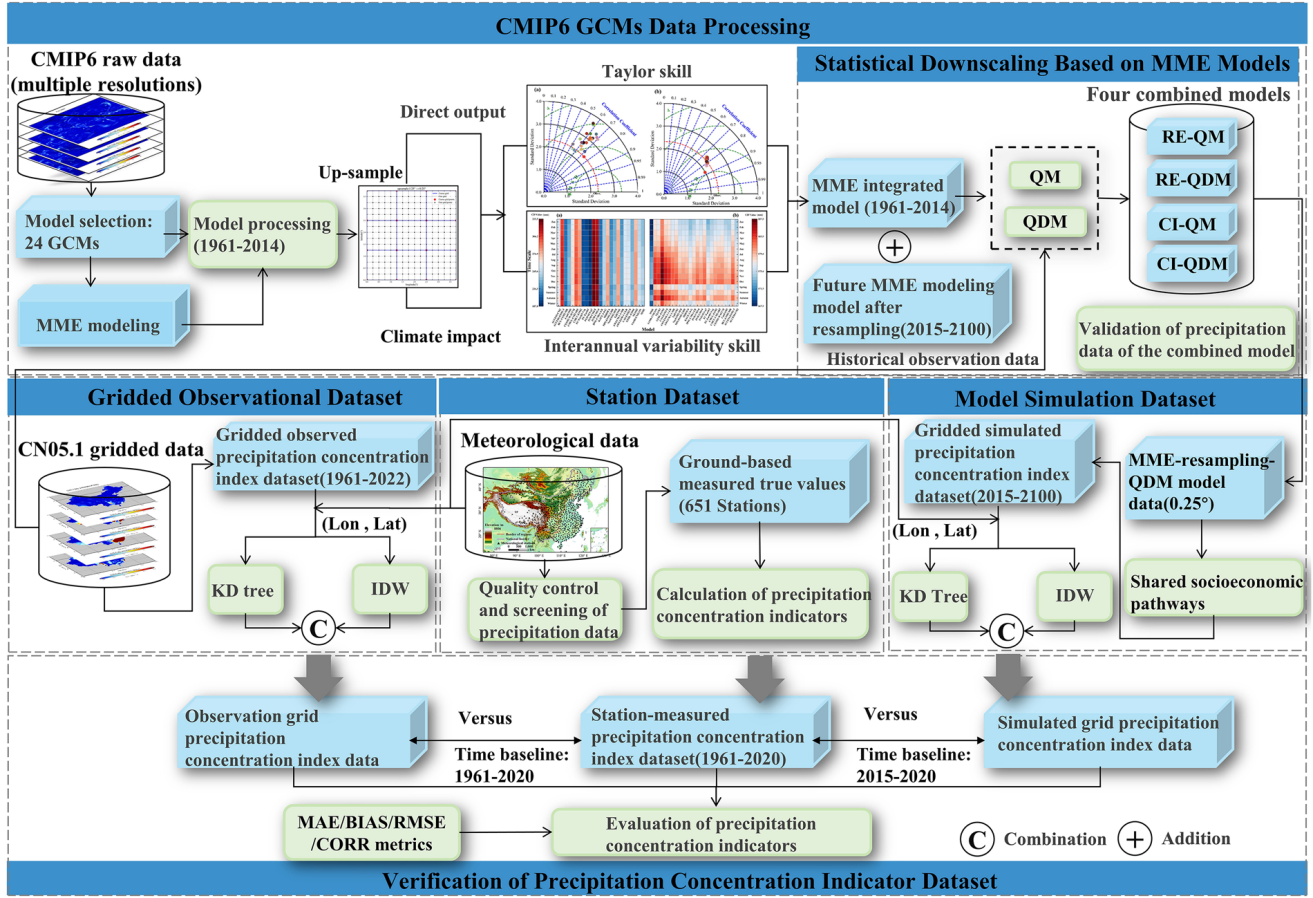
Flowchart for the preparation of a MPCID.

A precipitation concentration indicator dataset for mainland China was systematically constructed, integrating historical observations and future climate scenarios (Fig. 2). The process of multi-source data preprocessing and dataset acquisition is as follows: using the quality-controlled precipitation data from 651 stations as ground-based measurements to calculate the precipitation concentration indices dataset; adopting the CN05.1 gridded precipitation dataset (1961–2022) to calculate a reference gridded precipitation concentration indices dataset for supplementary spatial analysis and context, in order to mitigate the spatial sampling limitations of station observations; carrying out data preprocessing and screening on the original output data of multiple GCMs, selecting ensemble models that perform better under historical and future scenarios, conducting statistical downscaling under four frameworks on the ensemble model outputs to obtain corrected future precipitation data for different scenarios under the optimal framework, and calculating the simulated gridded precipitation concentration indices data based on the corrected precipitation data. The precipitation concentration products based on simulated grids are verified for their accuracy primarily by comparison with ground-station observation data. Comparisons with the CN05.1-based gridded products were provided for reference only, to illustrate spatial consistency. A multi-dimensional performance evaluation framework was established to systematically assess the reliability of the dataset: calculating basic error metrics such as mean absolute error (MAE) and root mean square error (RMSE) to quantify the differences between simulated and measured precipitation; adopting the Pearson correlation coefficient (CORR) to evaluate the linear association between simulated and measured precipitation concentration indicators; and simultaneously calculating the interannual variability skill (IVS) score and Taylor skill score (TS) to assess the ability of downscaled data to reproduce precipitation and provide a comprehensive evaluation. Crucially, the MME was constructed using daily-scale data from all GCMs. For GCMs with only monthly output, resampled daily data were incorporated solely for historical validation and MME averaging, but all precipitation concentration indicators were derived from the daily-scale MME data. This approach ensures that future-period DPCI evaluation is not limited by the availability of native daily GCMs output, as the MME provides a consistent daily basis for all indicators.

### Indicators derivation

PCD is a valid index that reflects the concentration of precipitation events and their distribution characteristics over a given period. Similarly, PCP identifies the period during which precipitation events are most concentrated. Zhang and Qian defined the concepts of concentration degree and concentration period, which were calculated using the principle of vector analysis^14^. In this method, total monthly precipitation is represented as a vector with both magnitude and direction, and the precipitation for the 12 months of the year is mapped onto a 360° circular scale^12,20,30^.

For the calendar mapping in this process, leap years are handled according to the actual calendar to retain the authenticity of date information; for climate model data adopting non-standard 360-day calendars, the corresponding angles are linearly mapped to the 0–360° range to achieve consistency with the standard calendar system. Cross-year phase differences are interpreted based on the minimal angular distance principle, ensuring the comparability of precipitation concentration period results across different data sources. It should be noted that for non-standard calendar data from individual CMIP6 models, we first converted them to the standard calendar format during preprocessing. Eventually, only the MME with standard calendar output, which exhibited superior simulation performance, was used for the calculation of precipitation concentration indicators.

**Fig. 3:**
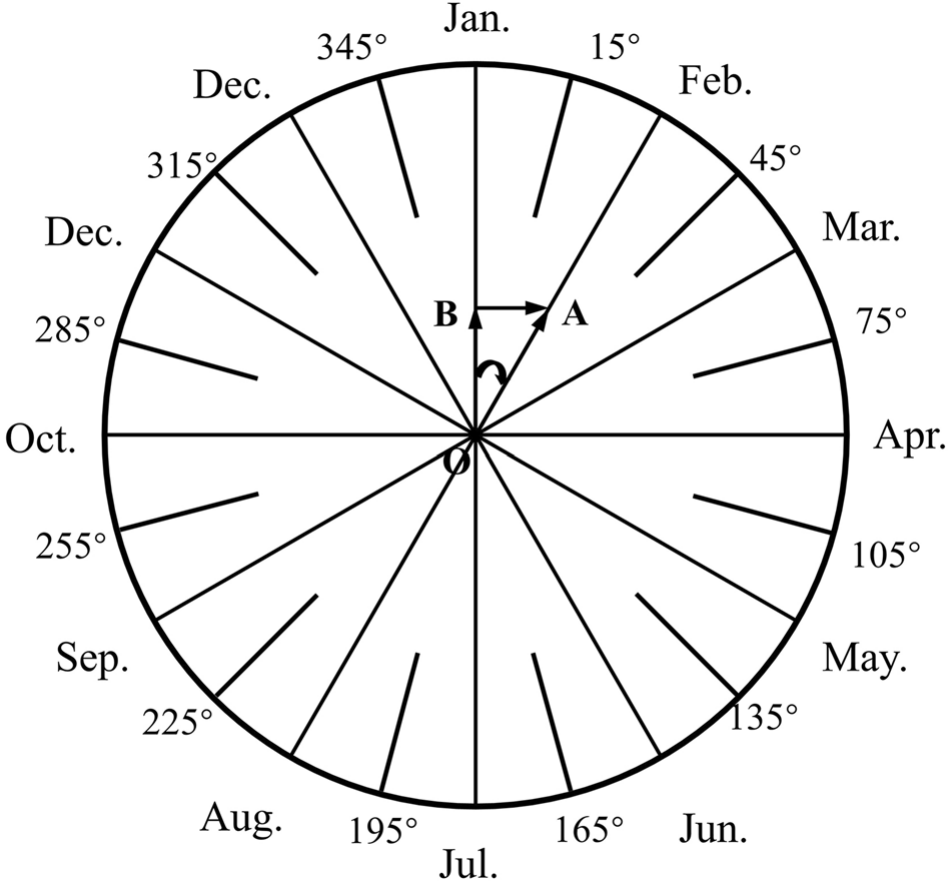
Corresponding relation between yearly PCP and month.

The magnitude of the vector corresponds to the amount of precipitation, while the direction indicates the time period of precipitation. This vector analysis method decomposes the monthly precipitation amount into components along the horizontal and vertical axes based on specific angles. The ratio of the cumulative precipitation component along the horizontal or vertical axis to the total annual precipitation is defined as the annual precipitation concentration. The azimuth of the resulting synthesized vector represents the period of concentration (Fig. 3). The calculation formula is as follows:

1 $${R}_{i}=\sum {r}_{ij},$$

2 $${R}_{{x}_{i}}=\mathop{\sum }\limits_{i=1}^{12}{r}_{ij}\sin \,{\theta }_{j},$$

3 $${R}_{{y}_{{\rm{i}}}}=\mathop{\sum }\limits_{i=1}^{12}{r}_{ij}\cos \,{\theta }_{j},$$

4 $$PCD=\frac{\sqrt{{R}_{{x}_{i}}^{2}+{R}_{{y}_{i}}^{2}}}{{R}_{i}},$$

5 $$PCP={\tan }^{-1}\left(\frac{{R}_{{x}_{i}}}{{R}_{{y}_{i}}}\right),$$

Schematic diagram of PCP calculation using vector analysis. The azimuth of the synthesized vector from monthly precipitation amounts represents the day of the year around which precipitation is most concentrated. The circular diagram illustrates how monthly precipitation is mapped to a directional vector to compute the PCP. To illustrate this vector method, consider a simplified, conceptual example for one year: if the majority of annual precipitation occurred entirely in July, the resultant vector would point towards the azimuth angle corresponding to mid-July (approximately 195°). In this extreme case, the PCD would approach 1, indicating perfect temporal concentration, and the PCP would be 195°, representing the timing of this concentration.

Where *R*_*xi*_ and *R*_*yi*_ are the components of total precipitation during the study period at the meteorological station in the x and y directions, respectively, calculated based on daily precipitation *r*_*ij*_ and the azimuth angle *θ*_*j*_ for each day in the study period. *R*_*i*_ is the total annual precipitation, *r*_*ij*_ is the daily precipitation, *i* is the year (*i* = 1961, 1962,…, 2100), and j refers to the day number within the year (*j* = 1, 2,…, 365 or 366). The azimuth angle *θ*_*j*_ for each day is mapped to a circle as follows: *θ*_*j*_ = (*j* - 0.5) * (360 / *T*_*year*_) degrees, where *T*_*year*_ is 365 or 366 depending on whether the year is a common year or a leap year. This ensures an even distribution of days across the 0–360° range. The resulting PCP is a circular variable with a range of 0–360°, representing the central day of precipitation concentration.

PCD represents the degree of concentration of total annual precipitation across the year, ranging from 0 to 1. When interpreting differences in PCP, the minimal angular distance across the 0 / 360° boundary is used. For example, the difference between a PCP of 5° and 355° is 10°, not 350°. When precipitation is highly concentrated within a few days or a specific time period, PCD reaches its maximum value of 1. When precipitation is evenly distributed across all days of the year, PCD reaches its minimum value of 0.

This study employed the DPCI proposed by Martin-Vide to quantify the temporal concentration of precipitation events^10^. The DPCI, which quantifies deviations between observed precipitation and a uniform distribution, was derived by analyzing the relationship between cumulative precipitation percentages and cumulative precipitation day percentages. The computational procedure was executed as follows:

Daily precipitation events with ≥0.1 mm were defined as valid precipitation days. This widely adopted threshold effectively excludes trace precipitation and focuses on measurable events that contribute substantially to hydrological processes^10,13^. Daily precipitation values were categorized into 1 mm interval bins and sorted in descending order based on precipitation intensity. The 1 mm binning strategy represents a standard compromise in DPCI applications, balancing computational efficiency with sufficient resolution to capture precipitation structure. The use of finer bins would increase sensitivity to drizzle and minor precipitation events, potentially inflating the concentration index by incorporating numerous low-impact days, while coarser bins might oversimplify the distribution and obscure important details of precipitation intensity. The total precipitation amount and number of precipitation days within each bin were cumulatively summed. These values were then converted into cumulative precipitation percentages (*Y*) and cumulative precipitation day percentages (*X*).

The nonlinear model was fitted to the cumulative data using the equation:

6 $$Y=X\cdot \exp [-b\cdot {(100-X)}^{c}]$$

where *X* is the cumulative percentage of precipitation days, *Y* is the cumulative percentage of precipitation, and parameters *b* and *c* were estimated via the Levenberg-Marquardt least-squares method to minimize the residual variance between observed and modeled values.

The area (*S*) between the modeled Lorenz curve (derived from the fitted equation) and the equidistribution line (45° line) was calculated as:

7 $$S=5000-{\int }_{0}^{100}X\cdot \exp [-b\cdot {(100-X)}^{c}]\begin{array}{l}dX\end{array}$$

The DPCI was computed as the ratio of *S* to the total area under the equidistribution line (5000):

8 $$DPCI=\frac{S}{5000}$$

This dimensionless index ranges from 0 to 1, where higher DPCI values indicate greater concentration of precipitation within fewer precipitation days.

MPCI was derived by De Luis based on the precipitation concentration index originally proposed by Oliver^6,31^. The modified index is described below.

9 $$MPCI=100\times \frac{(\mathop{\sum }\limits_{i=1}^{12}{{p}_{i}}^{2})}{{(\mathop{\sum }\limits_{i=1}^{12}{p}_{i})}^{2}}$$

Let *p*_*i*_ represent the monthly precipitation amount for month *i*. According to Eq. (9), if the annual precipitation is concentrated in a certain month, the MPCI equals 100. If the annual precipitation is evenly distributed across all 12 months, the MPCI approaches 8, which represents the minimum value. This establishes the theoretical range of MPCI from approximately 8 (uniform allocation) to 100 (complete concentration), which contrasts with the [0, 1] bounds shared by the PCD and DPCI indicators. MPCI is calculated on an annual scale to quantify the degree of precipitation concentration for a given year. In this study, the MPCI for a given year was calculated using the annual precipitation data for that specific year. The index can also be computed based on long-term average monthly precipitation, but that approach was not employed here.

### Statistical downscaling frameworks

Quantile Mapping (QM) and its enhanced variant, Quantile Delta Mapping (QDM), are probability distribution-based statistical downscaling frameworks designed to mitigate distributional biases between climate model outputs and observational data, thereby improving the reliability of regional-scale climate projections. While both methods share the theoretical foundation of probability distribution matching, they fundamentally diverge in their mechanisms for preserving climate change signals.

QM eliminates absolute quantile biases through direct distributional adjustment, as expressed by:

10 $${X}_{corr}(t)={F}_{obs}^{-1}({F}_{\mathrm{mod}.hist}({X}_{raw}(t)))$$

Where the raw model output *X*_raw_(*t*) represents the original precipitation simulation value of the climate model at a specific time t (unit: mm); *F*_*mod.hist*_ denotes the cumulative distribution function (CDF) of precipitation during the historical simulation period of the climate model (a baseline period matching observational data, dimensionless); and ${F}_{obs}^{-1}$ stands for the inverse cumulative distribution function of observed precipitation (dimensionless). This inverse function maps the quantiles of model output to the observational quantile space based on the statistical distribution characteristics of observed precipitation, so as to eliminate the absolute quantile bias of the model. Although QM effectively aligns model quantiles with observations through nonlinear transformation, it inherently overwrites the model-projected climate change signal (ΔQ) in future scenarios.

QDM preserves relative climate change signals by decoupling absolute bias correction from trend retention, implemented via a two-step algorithm:

Quantile delta factor calculation:

11 $$\Delta {Q}_{\mathrm{mod}.fut}(t)=\frac{{X}_{\mathrm{mod}.fut}(t)}{{F}_{\mathrm{mod}.hist}^{-1}(p(t))},where\begin{array}{l}p(t)\end{array}={F}_{\mathrm{mod}.hist}({X}_{\mathrm{mod}.fut}(t))$$

In this equation, Δ*Q*_*mod.fut*_(*t*) represents the relative climate change signal projected by the climate model for the future period (dimensionless), which is calculated based on the ratio of precipitation quantiles between the model’s historical simulation and future projection. It is used to quantify the magnitude of precipitation change in the future relative to the historical period. The definition of this variable refers to previous elaborations on the climate change signal retention mechanism in the QDM method, ensuring that the long-term change trends predicted by the model are not lost during the quantile adjustment process^32^.

Delta factor application:

12 $${X}_{corr}(t)={F}_{obs}^{-1}(p(t))\times \Delta {Q}_{\mathrm{mod}.fut}(t)$$

Here, ${F}_{obs}^{-1}(p(t))$ represents the model output after historical bias correction (unit: mm), which is obtained by applying the inverse cumulative distribution function of observational data to the historical simulation values, with the purpose of first eliminating the historical absolute bias of the model; the subsequent coupling with Δ*Q*_*mod.fut*_(*t*) realizes bias correction while retaining the climate change signal.

QM demonstrates strong empirical competency in marginal distribution alignment but introduces substantial overfitting risks to training periods, potentially distorting emergent climate signals^33^. Conversely, QDM preserves climatic fidelity through temporal change quotient retention, demonstrating particular utility in climate-driven extreme event characterization^34^. Both techniques require complete cumulative distribution function specification, introducing reliability constraints in data-scarce environments and non-stationary climates.

These approaches were selected based on demonstrated competency in resolving higher-moment distributional mismatches while preserving climate forcing trajectories^35^. Importantly, the framework facilitates localized parameterization without prior distribution assumptions, enhancing regional climate representativeness^36^.

To facilitate readers’ quick understanding of the symbols in the QM and QDM formulas and avoid ambiguity in variable interpretation, Table 2 systematically summarizes all core symbols involved in both methods, including their specific descriptions and corresponding units.

**Table 2:** Symbols and definitions for QM / QDM formulas.

Symbol	Description	Unit
${X}_{{\rm{corr}}}(t)$	Bias-corrected precipitation data at time t (applicable to both QM and QDM)	mm
${X}_{raw}(t)$	Raw precipitation output of climate models at time t (for QM)	mm
${X}_{\mathrm{mod}.{fut}}(t)$	Future precipitation simulation data of climate models at time t (for QDM)	mm
${F}_{\mathrm{mod}.hist}(\,\cdot \,)$	Cumulative distribution function of precipitation in model historical simulation period	Dimensionless
${F}_{\mathrm{mod}.hist}^{-1}(\,\cdot \,)$	Inverse cumulative distribution function of precipitation in model historical simulation period	Dimensionless
${F}_{obs}^{-1}(\,\cdot \,)$	Inverse cumulative distribution function of observed precipitation	Dimensionless
$p(t)$	Quantile of *X*_*mod.fut*_(*t*) in the historical model precipitation distribution (for QDM)	Dimensionless
$\varDelta {Q}_{{\rm{mod}}{\rm{.fut}}}(t)$	Quantile delta factor at time t, quantifying the relative change ratio of future model-projected precipitation to historical precipitation (for QDM)	Dimensionless

### Statistical methods

The methodological performance of precipitation downscaling and the accuracy of concentration indicators were assessed to quantify discrepancies between model-simulated data and ground-based observational datasets. Grid precipitation concentration products were systematically compared with ground station observations. Additionally, a multi-dimensional performance evaluation framework was implemented, comprising three clusters of validated metrics: basic error diagnostics, correlation-pattern similarity analyses, and integrated skill-variability assessments.

The Basic Error Metrics module incorporates two essential dimensional quantifiers, MAE and RMSE, to quantify the average magnitude of absolute errors. These indicators directly measure absolute deviations between model-simulated and observation-derived precipitation estimates. MAE serves as a direct quantifier of predictive accuracy with outlier robustness, whereas RMSE amplifies the weighting of extreme errors through its squared-error formulation^37^. A dedicated systematic Bias indicator was incorporated to diagnose persistent model overestimation / underestimation trends, providing pivotal references for bias-correction protocols^38^. This integrative strategy enabled comprehensive error characterization by simultaneously addressing systematic and stochastic biases, thus preventing partial assessments associated with isolated indicator applications^39,40^.

The CORR is used to evaluate the degree of linear association between the simulated and observed precipitation concentration indices, and can effectively capture / temporal fluctuation patterns^41^. It is important to note that the application of the linear Pearson correlation to the circular variable PCP has inherent limitations, as it does not account for the circular nature of the data (the proximity of 0° and 360°). In this study, the CORR for PCP is interpreted with caution, primarily as an indicator of the overall co-variation in temporal patterns, while the MAE and RMSE of the minimal angular distance provide a more geometrically appropriate measure of error.

The integrated skill and variability metrics group includes IVS and TS. IVS is specifically designed to measure the ability of downscaled data to replicate the inter-annual variability of precipitation concentration patterns. This metric is particularly important for assessing the model’s performance in capturing the variability that drives extreme hydrological years, such as droughts or floods^2^. IVS eliminates the influence of temporal phase alignment and instead focuses solely on the magnitude of inter-annual variability by utilizing standard deviation matching ratios. TS, based on correlation coefficients and standard deviation ratios, provides a more integrated assessment by simultaneously considering pattern similarity, amplitude, and variability consistency between simulated and observed datasets^39,42^.

## Data Records

MPCID is accessible via the permanent DOI (10.6084/m9.figshare.28656086)^22^. MPCID includes comma-separated values (CSV) files and gridded data products. The CSV files are organized into four folders, each corresponding to one precipitation concentration indicator. Each folder contains annual values of that indicator for individual stations from 1961–2020. The gridded data products are provided in Network Common Data Form (NetCDF) format, featuring a spatial resolution of 0.25° (~30 km). All gridded data use the WGS-84 geographic coordinate system. The historical period covers 1961–2022, and future projections cover 2015–2100 under four SSP scenarios (SSP1-2.6, SSP2-4.5, SSP3-7.0, and SSP5-8.5). The temporal coverage is continuous across these periods, and the standard Gregorian calendar is used. Missing values are encoded as NaN across all data files.

## Technical Validation

### Processing of historical data

Initial atmospheric circulation model outputs had low resolution and exhibited inconsistent resolutions across models, thus necessitating resampling to a uniform 0.25° × 0.25° grid for downstream applications. This resampling process employed CN05.1 observational data as a reference, applying bilinear interpolation to ensure spatial consistency across grids. To address systematic uncertainties and minimize biases, a multi-model ensemble averaging approach was adopted, assuming independent errors across GCMs. The reliability of precipitation concentration estimates is critically dependent on precipitation data quality, with higher observational correlations and reduced biases enhancing result robustness.

Given the inherent limitations of GCMs in resolving localized climate dynamics and their sub-optimal precipitation simulation performance, the Climate Impact (CI) bias-correction method was implemented. This method established statistical linkages between large-scale atmospheric variables and local climate parameters using historical observations, thereby mitigating systematic discrepancies between GCMs outputs and observed data^43^.

**Fig. 4:**
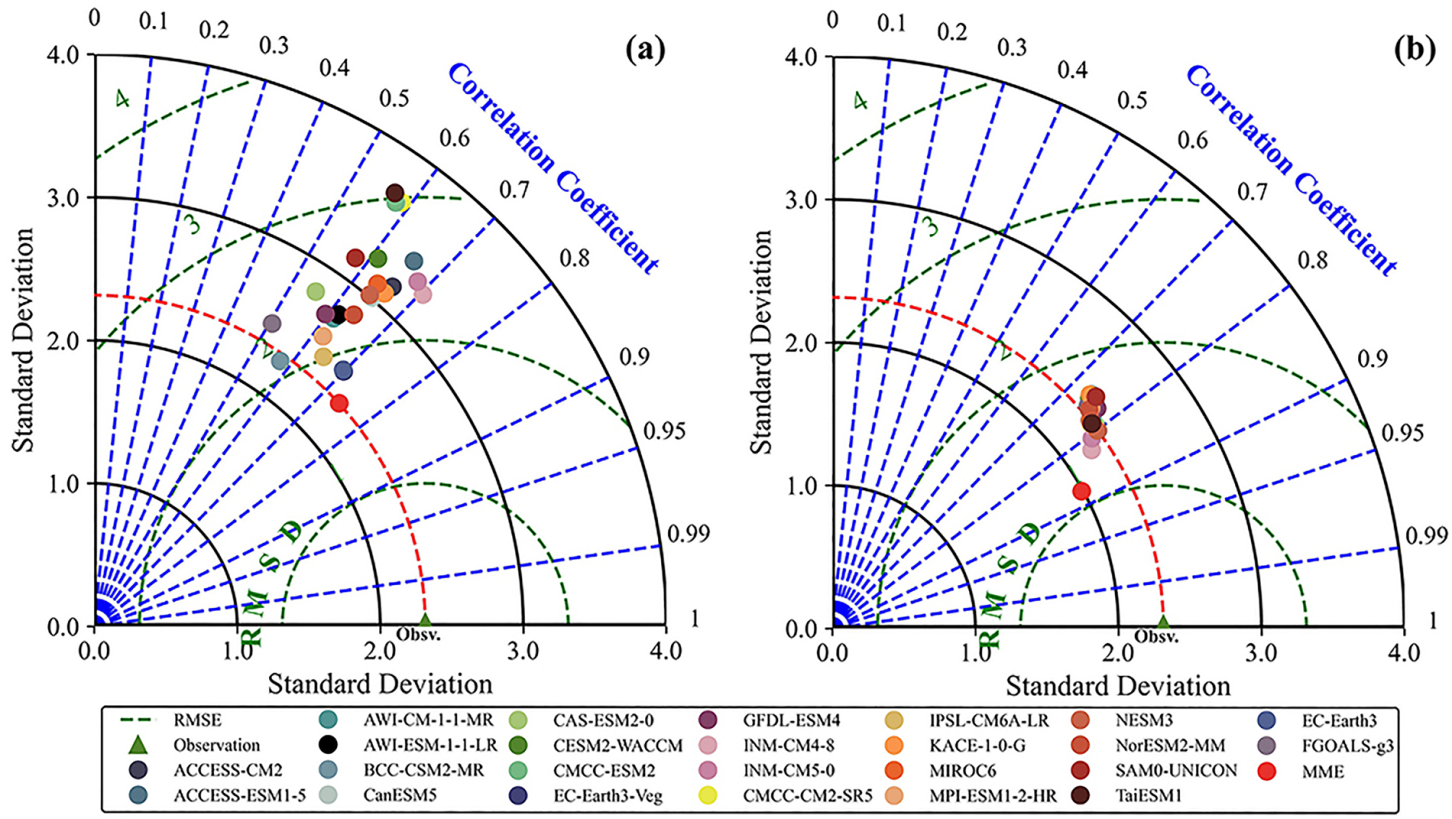
Taylor diagram of CMIP6 model versus observed precipitation from 1961 to 2014, (**a**) resampled, (**b**) CI applied after resampling. blue dashed lines in the plots indicate the correlation coefficients with the observed patterns; the green lines indicate the root-mean-square error of each CMIP6 pattern with respect to the reference value; the black lines indicate the standard deviation; and the observed values are the reference values of the observed patterns. The red circles correspond to the multi-model precipitation ensemble mean composite data; the name of each CMIP6 model and the corresponding label are shown in the upper right of the figure.

Table 3 presents quantitative evaluations of 24 GCMs monthly precipitation simulations from 1961 to 2014, comparing resampled (Re-Monthly) and CI-corrected (CI-Monthly) historical datasets. Resampled data revealed TS values for individual models ranging from 0.5136 (TaiESM1) to 0.8631 (EC-Earth3-Veg). In contrast, the MME achieved a markedly higher TS score of 0.9940, demonstrating superior overall accuracy. CI correction generally led to higher TS scores compared to resampled data, though the MME retained the highest overall accuracy despite a modest TS score decrease from 0.9940 to 0.9774. Lower IVS indicated enhanced interannual variability representation across GCMs after correction. Taylor diagrams showed a more robust MME accuracy through higher pattern correlations, with the resampled MME achieving a 0.74 correlation coefficient and showing lower standard deviation and RMSE than most individual models. CI correction increased the MME correlation coefficient to 0.88 while reducing both SD and RMSE (as shown in Fig. 4).

**Table 3:** Quantitative evaluation results of monthly precipitation simulations after bias correction from the 24 GCMs (1961–2014).

Model name	CI-Monthly		Re-Monthly		Δ*S*	Δ*IVS*
	S	IVS	S	IVS		
MME	0.9774	9.258 × 10^−2^	0.9940	2.410 × 10^−2^	0.0166	−6.848 × 10^−2^
INM-CM4-8	0.8908	1.019 × 10^−2^	0.7802	2.255 × 10^−2^	−0.0277	1.236 × 10^−2^
INM-CM5-0	0.8593	3.273 × 10^−3^	0.7407	2.365 × 10^−2^	−0.0014	2.038 × 10^−2^
AWI-ESM-1-1-LR	0.8502	1.372 × 10^−6^	0.6890	1.792 × 10^−2^	−0.0853	1.791 × 10^−2^
NESM3	0.8498	2.445 × 10^−6^	0.7025	4.833 × 10^−2^	−0.1426	4.833 × 10^−2^
CAS-ESM2-0	0.8331	6.936 × 10^−5^	0.5828	1.788 × 10^−3^	−0.1939	1.719 × 10^−3^
TaiESM1	0.8195	4.001 × 10^−6^	0.5136	1.090 × 10^−1^	−0.1315	1.090 × 10^−1^
CMCC-CM2-SR5	0.8193	2.333 × 10^−5^	0.5398	1.666 × 10^−1^	−0.0954	1.666 × 10^−1^
IPSL-CM6A-LR	0.8162	9.068 × 10^−5^	0.7649	1.302 × 10^−1^	−0.1273	1.301 × 10^−1^
EC-Earth3	0.8086	8.975 × 10^−5^	0.8579	1.037 × 10^−1^	−0.1509	1.036 × 10^−1^
EC-Earth3-Veg	0.8076	2.891 × 10^−7^	0.8631	1.394 × 10^−1^	−0.2734	1.394 × 10^−2^
MIROC6	0.8073	4.534 × 10^−9^	0.6875	2.833 × 10^−1^	−0.0931	2.833 × 10^−1^
CMCC-ESM2	0.8070	4.064 × 10^−5^	0.5326	2.851 × 10^−1^	−0.1045	2.847 × 10^−1^
ACCESS-ESM1-5	0.8023	4.626 × 10^−5^	0.6837	3.420 × 10^−1^	−0.0786	3.415 × 10^−1^
GFDL-ESM4	0.7893	6.247 × 10^−3^	0.6577	4.923 × 10^−1^	−0.0073	4.860 × 10^−1^
ACCESS-CM2	0.7820	3.725 × 10^−3^	0.7196	1.504 × 10^−1^	−0.1992	1.467 × 10^−1^
CanESM5	0.7771	1.308 × 10^−3^	0.7142	3.998 × 10^−1^	−0.0575	3.985 × 10^−1^
NorESM2-MM	0.7768	1.494 × 10^−3^	0.7239	3.564 × 10^−1^	−0.0893	3.549 × 10^−1^
MPI-ESM1-2-HR	0.7762	2.584 × 10^−3^	0.7072	5.290 × 10^−1^	−0.0355	5.264 × 10^−1^
AWI-CM-1-1-MR	0.7715	8.072 × 10^−3^	0.6880	4.764 × 10^−1^	−0.1444	4.683 × 10^−1^
FGOALS-g3	0.7639	2.242 × 10^−3^	0.5342	6.137 × 10^−1^	−0.0802	6.115 × 10^−1^
SAM0-UNICON	0.7561	1.344 × 10^−2^	0.5878	3.991 × 10^−1^	−0.1683	3.857 × 10^−1^
CESM2-WACCM	0.7462	7.052 × 10^−3^	0.6271	8.737 × 10^−1^	−0.2137	8.666 × 10^−1^
BCC-CSM2-MR	0.7408	1.123 × 10^−2^	0.6392	9.017 × 10^−1^	−0.2010	8.904 × 10^−1^
KACE-1-0-G	0.7384	9.778 × 10^−3^	0.7238	9.325 × 10^−1^	−0.2248	9.228 × 10^−1^

**Fig. 5:**
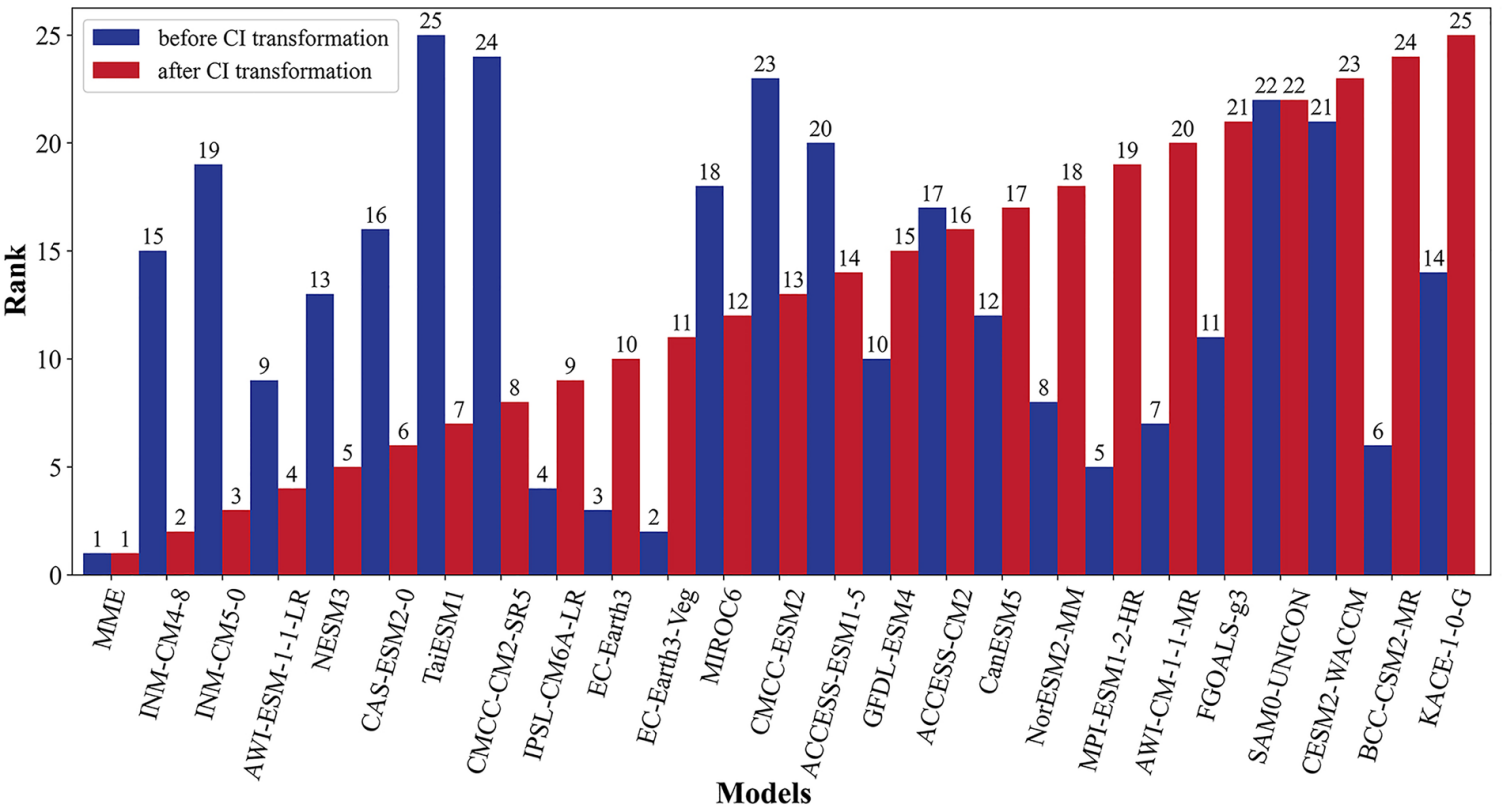
Comparison of model rankings before and after CI transformation.

**Fig. 6:**
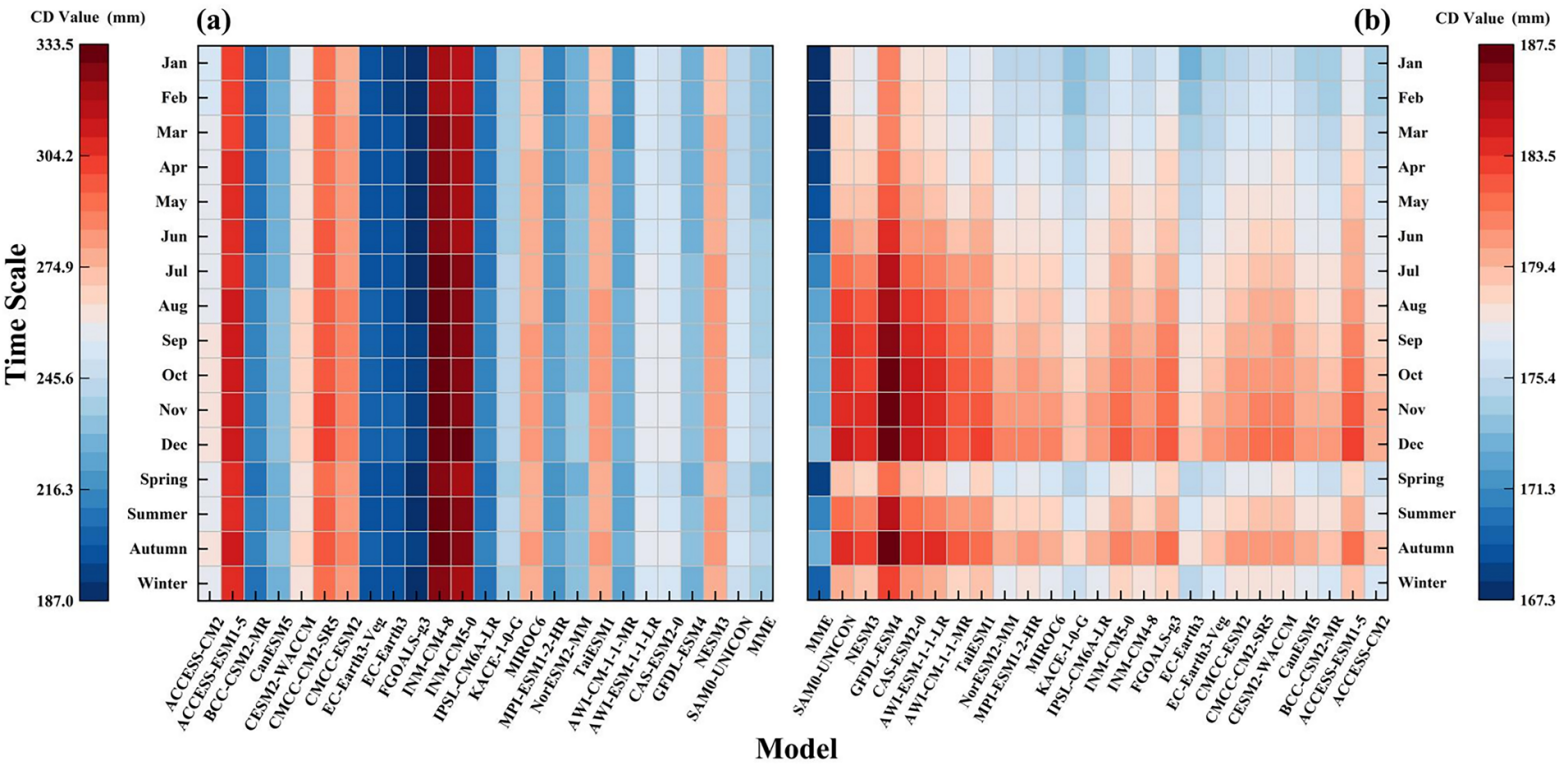
Portrait diagrams showing the intra-annual distribution of the CD values of precipitation between the GCMs and CN05.1 during 1961–2014 at different time scales.

Composite rankings in Fig. 5 compares model performance before and after CI correction using differential TS and IVS metrics. Models with poor resampling performance showed improved predictive skill after CI correction, whereas the MME experienced a slight reduction in accuracy. Despite a slight adjustment in its metrics, the MME maintained its position as the top-performing dataset after correction, justifying its retention for subsequent analyses. Seasonal simulations utilizing cumulative departure (CD) analysis in Fig. 6 revealed that resampled data had systematic overestimation biases. Winter precipitation values decreased after correction, summer biases were significantly reduced compared to original outputs, and spring / autumn simulations achieved balanced biases with minor overestimation.

Performance ranking of GCMs before and after applying the CI bias correction method. Model rankings are based on a composite of TS and IVS scores for monthly precipitation simulations against CN05.1 observations over the period 1961–2014.

### Downscaling and evaluation of future projected data

To ensure the scientific rigor of QDM-based bias correction and subsequent validation, the multi-source data were divided into three functional periods based on the core principle of QDM^32^. The historical quantile construction period corresponds to 1961–1999, where 1961–1999 GCM historical outputs were paired with 1961–1999 *in-situ* / CN05.1 observational data to fit the cumulative distribution functions (CDFs) of historical precipitation. This step establishes the statistical relationship for calculating the delta factor, which quantifies the relative change in precipitation projected by GCMs under future scenarios and is essential for QDM to preserve climate change signals. The baseline assessment period corresponds to 2000–2022 and serves as the validation window, as it is the only segment where QDM-corrected future data for 2000–2100 overlaps with contemporaneous observational data from 1961–2022. This 23-year window is sufficiently long to capture interannual climate variability, enabling objective evaluation of correction accuracy. Additionally, this period covers the early phase of SSPs projections (2015–2022), so validating the corrected 2000–2022 data ensures the reliability of the corrected 2015–2100 SSPs data for subsequent analyses. The future application period corresponds to 2023–2100, the target period for bias correction. Here, QDM-corrected data calibrated using 1961–1999 CDFs and validated against 2000–2022 observations were used to analyze future precipitation concentration trends under SSP scenarios. The 1961–1999 period was excluded from the baseline assessment to avoid circular reasoning, as it was dedicated to CDF fitting. Using the independent 2000–2022 period for validation ensures the robustness of the results.

Deriving precipitation concentration indicators for climate prediction requires statistically robust modeled precipitation datasets. In the methodology section, we systematically evaluated how CDF characteristics in the model output influence the application of QM and QDM bias correction. Historical observations play a dual role, establishing calibration benchmarks through CDF construction and preserving climate change signals during bias correction. Given the crucial role of historical data in balancing accuracy and signal preservation, we evaluated two processing methods for QM and QDM, namely direct historical output resampling and CI-enhanced resampled data. Four downscaling frameworks were implemented, including resample-QM (QM on resampled outputs), resample-QDM (QDM on resampled outputs), CI-QM (CI-optimized data with QM), and CI-QDM (CI-optimized data with QDM).

**Fig. 7:**
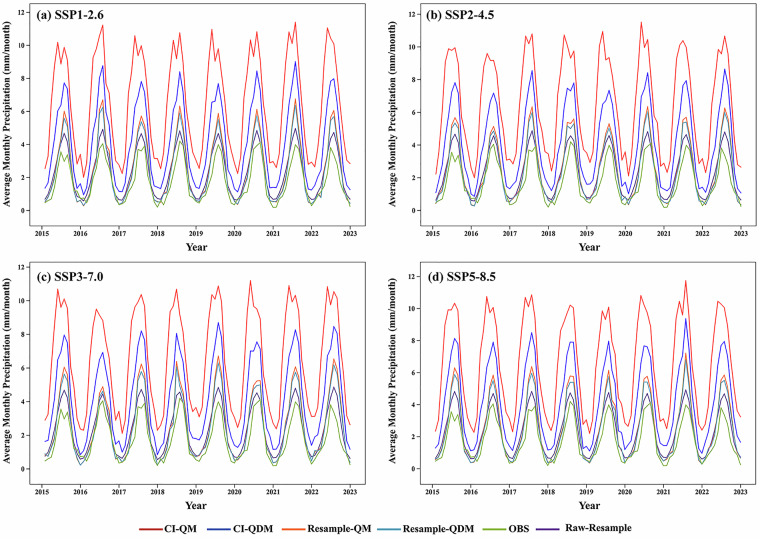
Monthly mean precipitation (unit: mm) of mainland China under different downscaling frameworks and SSP scenarios, compared with reference datasets (2015–2022). Notes: The y-axis represents monthly mean precipitation (unit: mm); the x-axis represents years (near-future validation period). Comparison objects include: four downscaling frameworks (resample-QM: QM applied to raw resampled CMIP6 data; resample-QDM: QDM applied to raw resampled CMIP6 data; CI-QM: QM applied to CI-corrected CMIP6 data; CI-QDM: QDM applied to CI-corrected CMIP6 data); two reference datasets (OBS: CN05.1 observational gridded data, used as ground truth; raw-resample: raw resampled CMIP6 data without bias correction).

Four downscaling frameworks were executed to generate validation data for the period of 2000–2022. An auxiliary validation baseline (“raw sample”) was introduced to evaluate the correction results from multiple dimensions. The raw sample was derived from the GCMs output of future projections, which was only resampled to ensure consistent resolution without undergoing statistical correction. Fig. 7 displayed the distinctly different monthly mean precipitation patterns of the four downscaling frameworks during 2015–2022. The CI-integrated framework exhibited greater volatility in wet season months, while less variability was observed in the resample-integrated framework. This indicated that the CI method amplified precipitation variability, leading to overestimation, whereas the resample-integrated framework more accurately captured precipitation trends in both rainy and dry seasons. Comparisons between the models and observed data highlighted that the resample-QDM approach was characterized by smaller errors and closer alignment with trends.

**Fig. 8:**
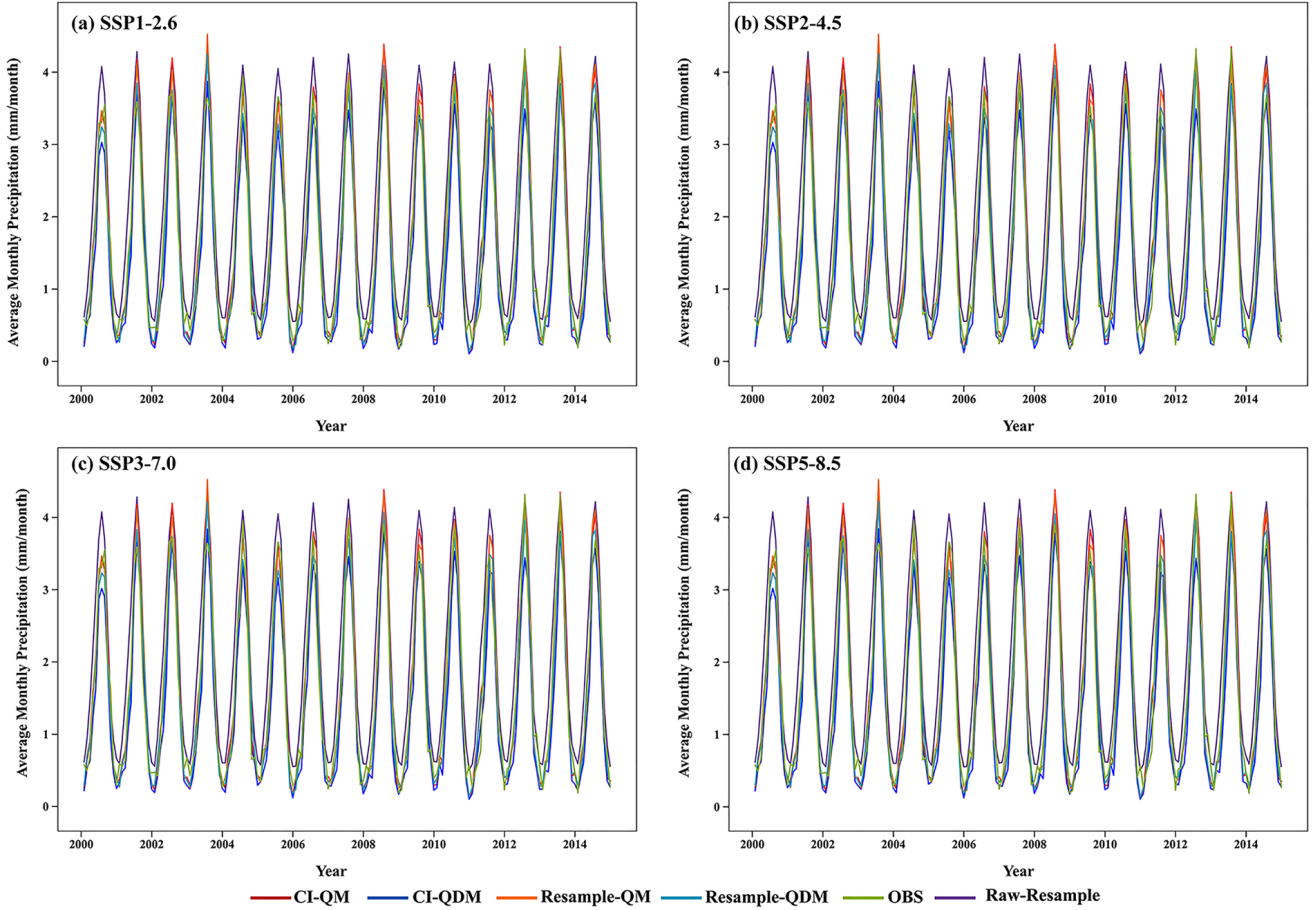
Monthly mean precipitation trends for the SSPs scenario with different downscaling configurations versus CN05 data and raw resampled outputs (2000 to 2014).

Numerically, the precipitation values of the corrected data from each method for 2015–2022 were slightly overestimated when compared to observed data, whereas the corrected data for 2000–2014 (Fig. 8) showed smaller deviations from observations, which might be attributed to the inherent systematic bias in model scenarios. During the 2015–2022 validation period, CI-QM had the highest mean monthly precipitation values among all methods, with significant deviations from both observed and original resampled datasets. In contrast, resample-QDM and raw-sampling frameworks were found to be closer to reference observations. The mean monthly precipitation derived from CI-QDM and resample-QM was moderate. The CI-integrated framework was prone to precipitation overestimation and exhibited greater volatility at extreme values, while non-CI methods maintained smoother trends, particularly in rainy season months.

Table 4 systematically evaluated four statistical downscaling configurations across different SSP scenarios. Validation against CN05.1 observational data revealed notable methodological differences between two key periods, the historical baseline (2000–2014), representing observed climatic conditions, and the scenario-based near-future period (2015–2022), which served as a proxy for early-stage climate projections. During the historical baseline, the CI-QM method exhibited the lowest performance under the SSP1-2.6 scenario, with significantly higher error metrics and a considerable positive bias. In contrast, the resample-QDM configuration achieved the best accuracy, showing minimal error values. Under SSP2-4.5 conditions, resample-QDM maintained its precision advantage (MAE = 1.147, RMSE = 2.158, BIAS = 0.625), while CI-QM presented increased error levels (MAE = 5.093, RMSE = 7.920) and consistent positive bias (BIAS = 4.677). Correlation analysis further underscored the superiority of resample-QDM (CORR = 0.863), which retained relatively strong correlation metrics compared to the considerably weaker performance of CI-QM (CORR = 0.518). This trend continued under SSP3-7.0, where resample-QDM effectively reduced errors relative to baseline configurations (MAE = 1.152, RMSE = 2.207), demonstrating consistent error suppression capabilities. In the SSP5-8.5 scenario, characterized by intensified climate forcing, resample-QDM sustained its high performance (MAE = 1.166, RMSE = 2.229), whereas CI-QM exhibited substantially increased errors (MAE = 5.124, RMSE = 7.957) and a pronounced bias (BIAS = 4.750). Comparisons across all scenarios identify resample-QDM as the most robust configuration, with consistently low error values and minimal bias variation.

**Table 4:** Comparative performance analysis of downscaling models against CN05.1 data under SSP scenarios.

Scenarios	data	Historical validation (2000–2014)				Near-future validation (2015–2022)			
		MAE	RMSE	BIAS	CORR	MAE	RMSE	BIAS	CORR
SSP1-2.6	CI-QDM	0.770	1.483	−0.212	0.887	2.749	4.355	2.359	0.775
	CI-QM	0.881	1.565	0.024	0.870	5.097	7.890	4.715	0.530
	resample-QDM	0.773	1.489	−0.045	0.874	1.176	2.292	0.684	0.869
	resample-QM	0.816	1.569	0.037	0.867	1.278	2.499	0.803	0.861
	raw-resample	0.954	1.604	0.433	0.844	1.116	1.850	0.562	0.839
SSP2-4.5	CI-QDM	0.772	1.491	−0.214	0.887	2.714	4.308	2.288	0.768
	CI-QM	0.881	1.565	0.025	0.870	5.093	7.920	4.677	0.518
	resample-QDM	0.773	1.495	−0.046	0.875	1.147	2.158	0.625	0.863
	resample-QM	0.816	1.569	0.037	0.864	1.234	2.313	0.739	0.854
	raw-resample	0.954	1.604	0.433	0.844	1.121	1.854	0.559	0.836
SSP3-7.0	CI-QDM	0.770	1.484	−0.213	0.887	2.745	4.381	2.315	0.763
	CI-QM	0.811	1.565	0.024	0.870	5.101	7.966	4.680	0.514
	resample-QDM	0.772	1.488	−0.046	0.874	1.152	2.207	0.618	0.865
	resample-QM	0.816	1.569	0.037	0.867	1.254	2.394	0.738	0.856
	raw-resample	0.954	1.604	0.433	0.844	1.125	1.871	0.554	0.835
SSP5-8.5	CI-QDM	0.769	1.481	−0.217	0.887	2.769	4.372	2.388	0.772
	CI-QM	0.811	1.565	0.024	0.870	5.124	7.957	4.750	0.531
	resample-QDM	0.771	1.486	−0.050	0.875	1.166	2.229	0.702	0.866
	resample-QM	0.816	1.569	0.037	0.867	1.272	2.425	0.826	0.857
	raw-resample	0.954	1.604	0.433	0.844	1.118	1.851	0.569	0.836

The baseline assessment period (2000–2022) is selected based on the principle of QDM, as it is the only window where corrected future data and contemporaneous observations overlap.

While the CI-QM method demonstrated reasonable performance during the 2000–2014 baseline period across all SSPs, its limitations became critical in projecting the 2015–2022 near-future period. Standard QM adjusts variables to statistically match historical distributions but disregards climate change signals in future scenarios. When applied to 2015–2022 projections, its rigid distribution alignment amplified positive precipitation biases by forcing future extremes into historical quantiles. By contrast, QDM preserves climate change deltas during quantile adjustments. This explains the sustained accuracy of resample-QDM, as it respects scenario-driven trends rather than overfitting historical patterns. However, the superior baseline-period performance of QM confirms its effectiveness for historical calibration, where no trend extrapolation is required. The limitations of CI-integrated frameworks originate primarily from the computational framework of the CI method. The precipitation scaling mechanism facilitates geometric amplification of interpolation uncertainties via multiplier propagation, exacerbating errors during the quantile mapping process when applying historical distributions to future projections. Wet-season precipitation tails are overestimated, leading to elevated RMSE values. Furthermore, daily value scaling disrupts precipitation autocorrelation structures, introducing spurious wet-dry fluctuations that negatively affect correlation metrics.

In summary, while the resample-QDM method demonstrates superior overall performance, all downscaling outputs, particularly those from CI-integrated methods, exhibit a positive bias in the upper quantiles of precipitation. This finding advises a cautious interpretation of indicators like DPCI and MPCI that are sensitive to extreme precipitation values, and underscores the importance of considering the quantile-dependent biases revealed in this validation.

**Fig. 9:**
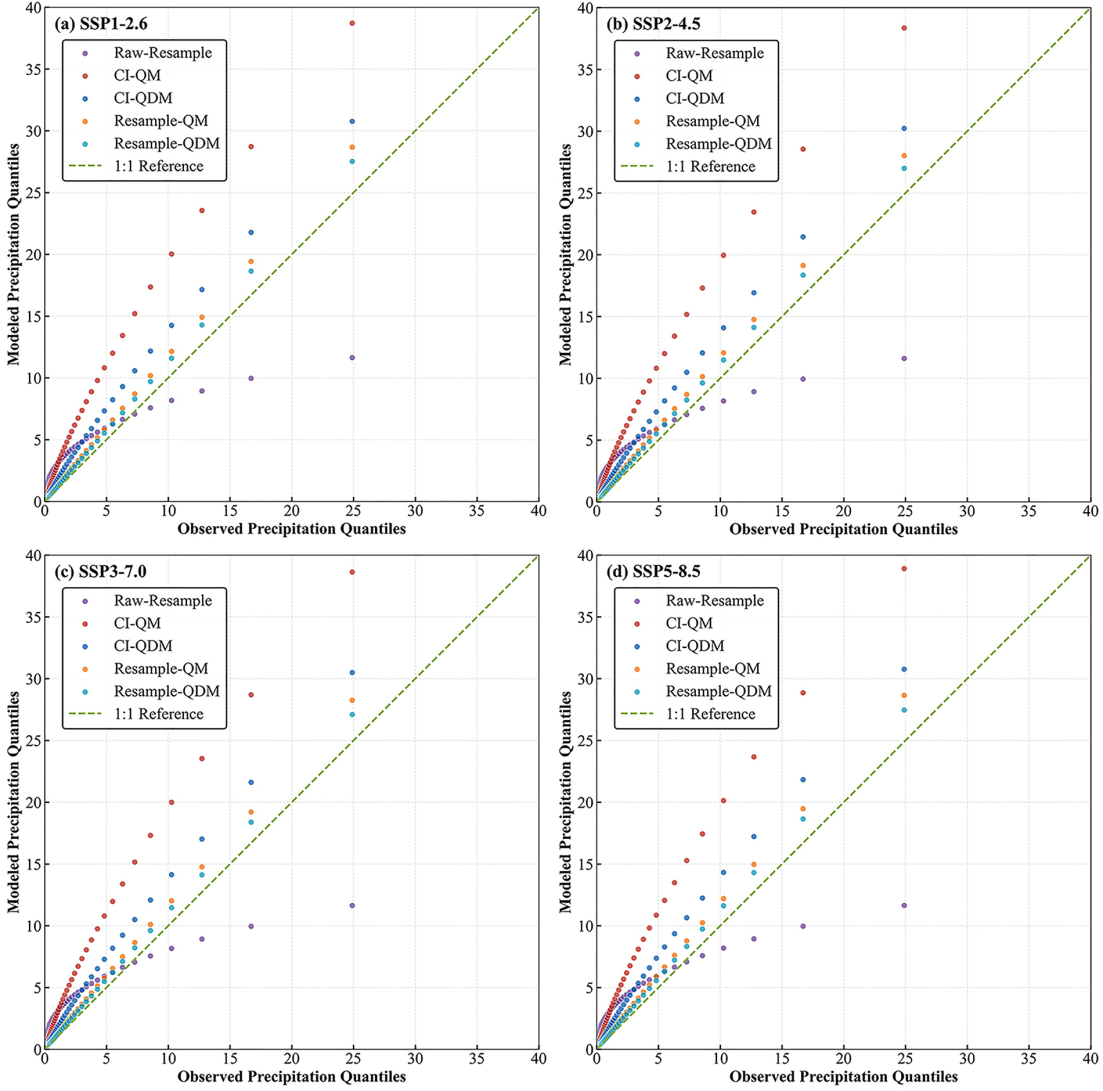
Quantile-Quantile (Q-Q) plots evaluating downscaled precipitation against observations. The distribution of daily precipitation from different downscaling methods under each SSP scenario is compared to the CN05.1 observational dataset for the historical validation period (2000–2022). The 1:1 line represents a perfect match.

To evaluate the reliability of downscaled precipitation data from resampled CMIP6 datasets via quantile mapping, Fig. 9 presents quantile-quantile (Q-Q) distributions between different downscaling methods and observation or resampled benchmarks across four emission scenarios (SSP1-2.6 to SSP5-8.5). Cross-scenario Q-Q plots corrected by the QDM method exhibit a uniform trend.

CI-QM and CI-QDM methods align closely with observations in lower-to-middle precipitation quantiles but systematically overestimate extreme precipitation at high percentiles; CI-QM shows slightly larger overestimation biases than CI-QDM. resample-QM and resample-QDM methods significantly improve the consistency across the full quantile range, and the corrected resampled data show better matching with observations in the high precipitation quantile range while exhibiting only minor deviations at extreme values. raw-resample method consistently underestimates mid-to-high precipitation quantiles in all scenarios, highlighting its inherent limitations in simulating extreme precipitation intensities.

However, it is important to note a systematic tendency for positive bias in the upper-tail extremes across all downscaling methods. This overestimation of very high precipitation values is most pronounced in the CI-integrated frameworks (CI-QM and CI-QDM). Consequently, users should exercise caution when utilizing the dataset, particularly for analyses that heavily rely on the accurate representation of extreme precipitation quantiles. Cross-referencing conclusions with the quantile behavior described here is strongly recommended.

### Trends in precipitation concentration indicators

**Fig. 10:**
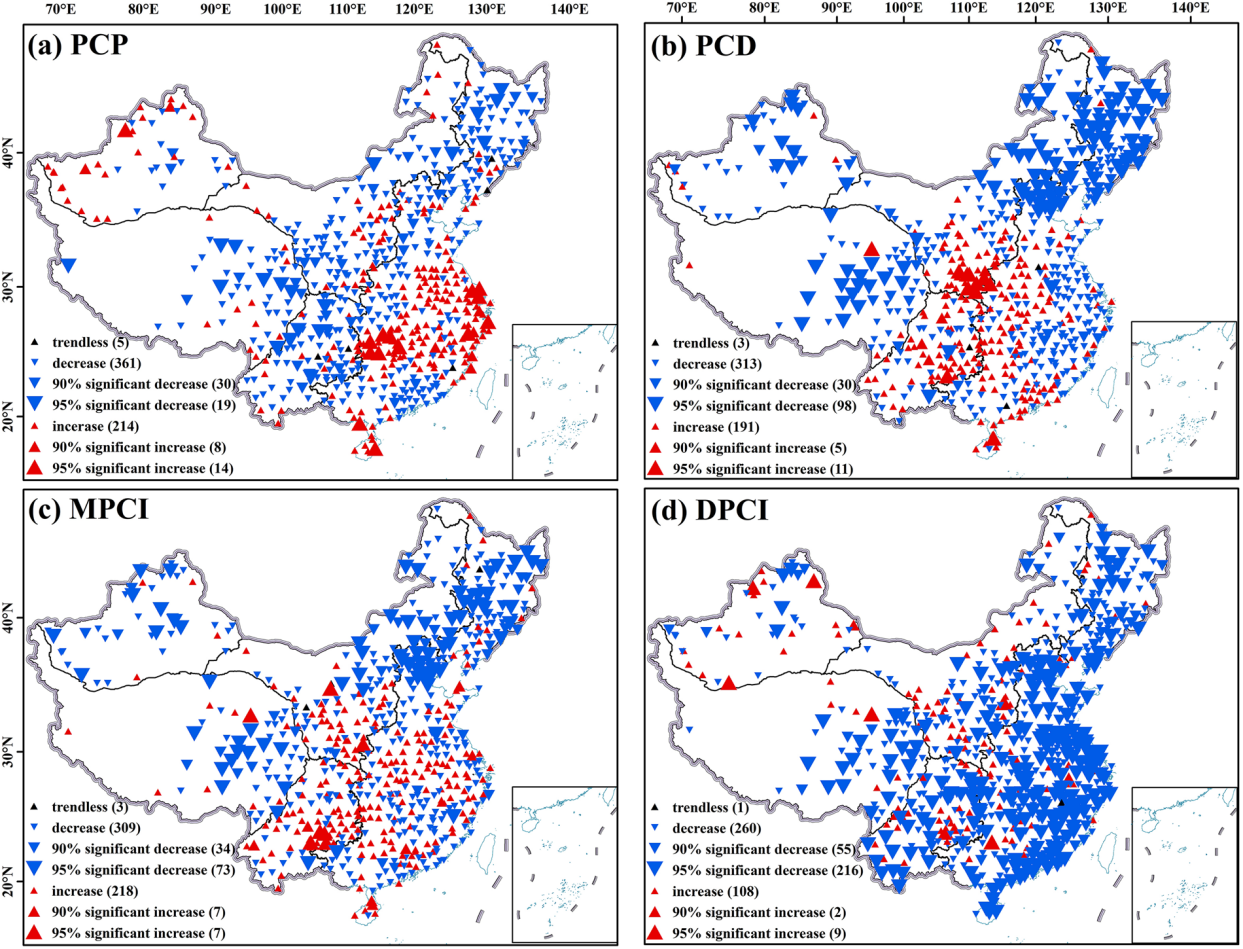
Interannual trends of precipitation concentration indicators based on data from meteorological stations, (**a**) PCP trends, (**b**) PCD trends, (**c**) MPCI trends, (**d**) DPCI trends.

**Fig. 11:**
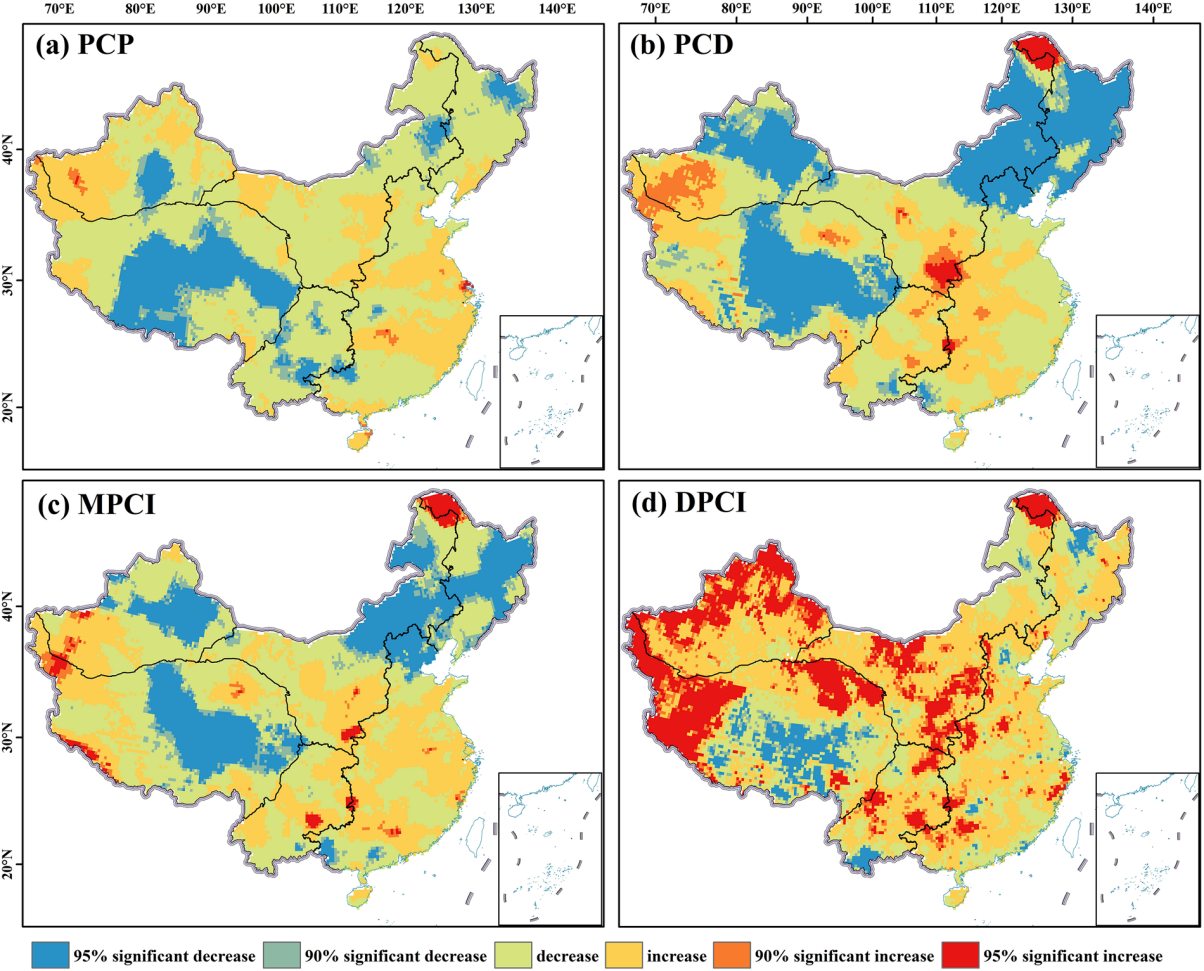
Inter-annual trends of precipitation concentration indicators based on grid data from CN05.1, (**a**) PCP trends; (**b**) PCD trends; (**c**) MPCI trends; (**d**) DPCI trends.

Analysis of precipitation concentration indices across mainland China reveals both coherent spatiotemporal patterns and scale-dependent divergences between station-based (Fig. 10) and gridded (Fig. 11) datasets.

Both datasets consistently show a significant advance (decrease) in PCP in central TP and NPM. This reflects regulatory effects of monsoon systems and subtropical high configurations on rainy season onset. PCD and MPCI decline notably in NPM, central TP and central XJ, indicating homogenized temporal distribution and mitigated extreme event risks. Meanwhile, increasing trends in Southwest China, eastern coast, and plateau margins link to enhanced convective activity or delayed monsoon phases, elevating extreme precipitation hazards.

Scale-dependent differences in DPCI are pronounced. Station data show negative trends in over 80 percent of sites, supporting the notion of homogenized daily precipitation and reduced extremes. Conversely, gridded data exhibit significant positive trends in northern XJ, western TP, and central China, indicating intensified daily-scale extremes driven by mesoscale convective system changes. This divergence stems from the interpolation characteristics of gridded data. Higher spatial heterogeneity in interpolated daily precipitation may amplify local extreme signals, whereas *in-situ* station measurements directly reflect ground-truth trends.

This comparison clarifies the fundamental boundary between station-based and grid-based trend analyses. The gridding process, by its nature, can either amplify localized extreme signals through spatial inference or smooth them out due to the influence of neighboring stations. Therefore, the station and grid perspectives are complementary but not fully equivalent. The station data provide a direct, point-based account of changes, while the gridded data offer a spatially continuous interpretation that may enhance regional patterns but should be interpreted with an understanding of the underlying interpolation methodology, especially in areas of complex topography or sparse station coverage.

Figures 10, 11 collectively highlight complex terrain zones such as eastern TP and monsoon-sensitive regions such as southeastern coast as core areas for precipitation concentration indices changes. The signals from station and gridded data are complementary: stations capture discrete ground-truth trends, while grids resolve fine-scale spatial heterogeneities.

**Fig. 12:**
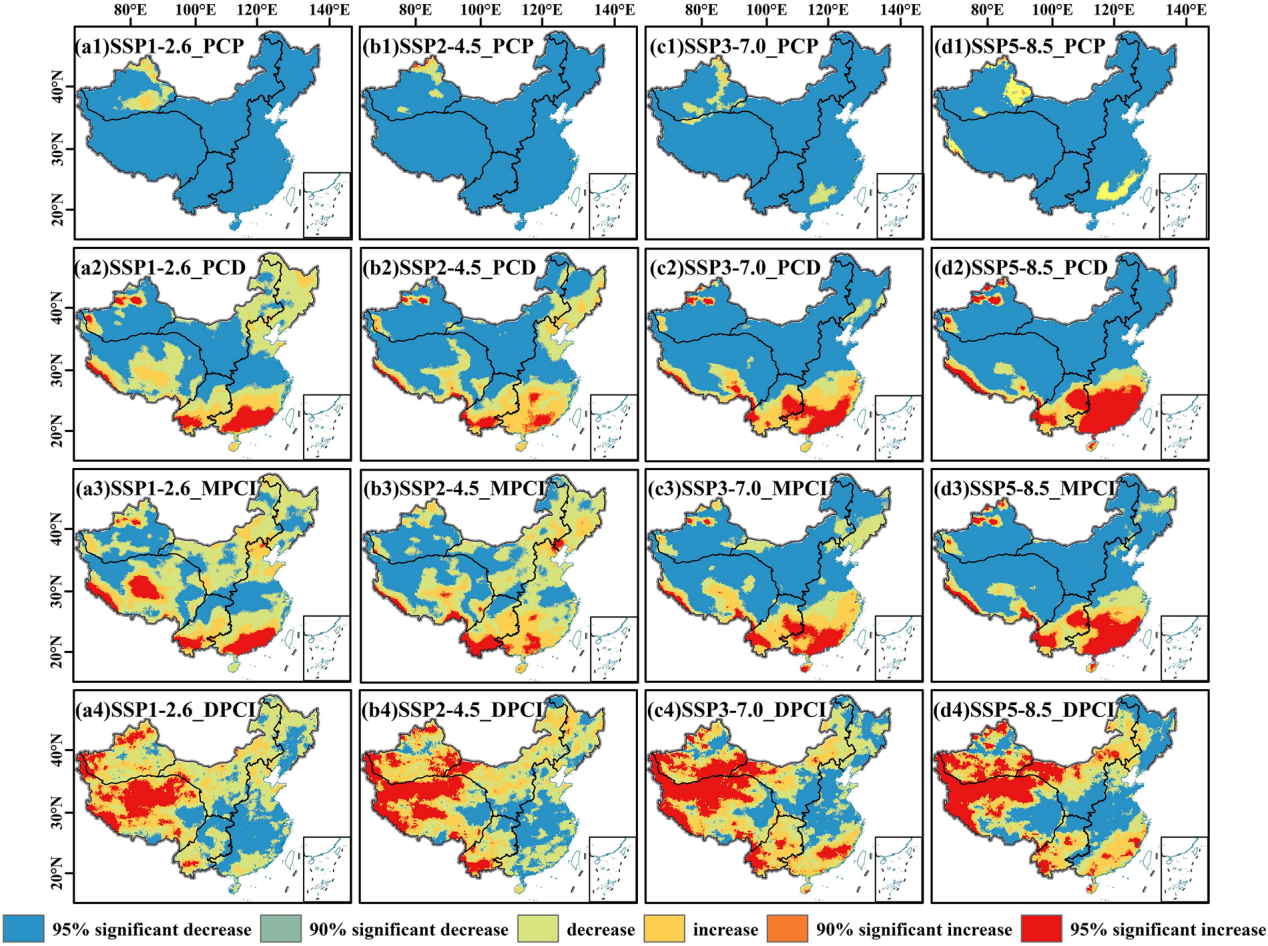
Projected future trends of precipitation concentration indicators under SSP scenarios. Spatial distribution of Sen’s slope trends for (**a**) PCD, (**b**) PCP, (**c**) DPCI, and (**d**) MPCI, calculated from the resample-QDM downscaled CMIP6 data for the period 2015–2100.

Under future climate scenarios, precipitation regimes over mainland China exhibit distinct spatial reorganizations, with notable variations across latitudinal and longitudinal gradients (Fig. 12). Low- to medium-emission pathways induce a broad shift toward earlier precipitation phases, characterized by widespread negative trends in precipitation concentration metrics. Exceptions include northern XJ and western TP, where localized positive trends suggest delayed onset of wet seasons, possibly linked to regional atmospheric circulation adjustments. High-emission scenarios intensify precipitation concentration in coastal and alpine regions. Eastern coastal zones witness enhanced precipitation clustering, driven by temperature-induced increases in vapor transport and monsoon dynamics, while inland plains experience attenuated extremes and more homogenized precipitation distributions. The Tibetan Plateau and western mountainous areas show amplified temporal clustering of precipitation, aligning with accelerated hydrological cycling under warming conditions.

Latitudinally, southern China under high emissions exhibits significant increases in precipitation concentration and extreme event magnitudes, contrasting with northern regions that show mixed trends, influenced by competing effects of monsoon shifts and moisture availability. Longitudinally, coastal-to-inland gradients in precipitation concentration intensify, with the southern seaboard emerging as a hotspot for concentrated extreme events, while western arid zones maintain relatively stable regimes except for localized enhancements in mountainous subregions. These patterns highlight climate change-driven disparities in precipitation regimes, with high-emission pathways exacerbating spatial heterogeneities in both intensity and distribution of precipitation events.

### Performance evaluation of precipitation concentration indicators

**Fig. 13:**
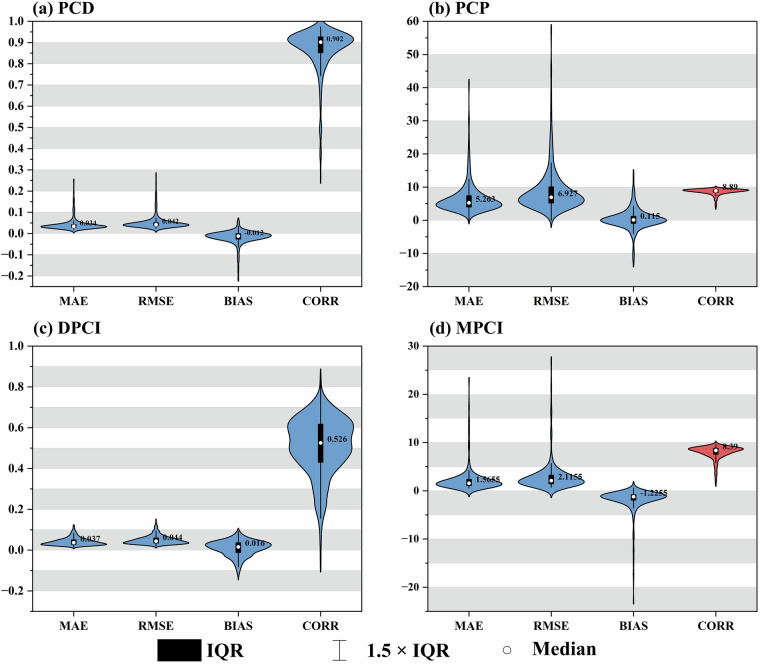
Evaluation of the four precipitation concentration indicators by comparing the gridded observational dataset (CN05.1) against ground station observations over the historical period (1961–2020). The sample size for each indicator is based on 651 stations over 60 years (1961–2020), resulting in 39,060 data points per indicator. The violin plots show the distribution of errors (MAE, RMSE, BIAS) and correlation for each indicator. In the boxplot elements within each violin, the black rectangles represent the interquartile range (IQR), thin black lines indicate the 1.5 × IQR range (whiskers), and white dots denote the median values. The red violin outlines are scaled by a factor of 10 to accentuate their morphological features.

Figure 13 presented the statistical metrics of four precipitation concentration indicators systematically evaluated using observation grid data, with violin plots visualizing their distributional characteristics. Among the evaluated metrics, PCD exhibited superior global consistency with the lowest MAE (0.034) and RMSE (0.042), while the correlation coefficient (CORR = 0.902) neared the theoretical maximum. These findings confirmed its strong capacity to characterize spatial heterogeneity of precipitation. Despite a marginal underestimation tendency (BIAS = −0.012), the statistically insignificant deviation confirmed the validity of PCD as a robust precipitation concentration metric. DPCI maintained better error control (MAE = 0.037, RMSE = 0.044) with minimal systematic bias (BIAS = 0.016), yet its low correlation (CORR = 0.526) revealed inherent limitations in modeling daily precipitation variability. MPCI displayed moderate correlation (CORR = 0.839) at monthly scales, but substantial errors (MAE = 1.565, RMSE = 2.115) and negative bias (BIAS = −1.225), indicating low sensitivity to extreme monthly precipitation. PCP showed a slight positive bias (BIAS = 0.115) and elevated errors (MAE = 5.263, RMSE = 6.927), mainly attributed to temporal misalignment of rainy season peak identification. Importantly, PCP retained high correlation (CORR = 0.885), suggesting systematic biases primarily affect its phase prediction reliability rather than temporal pattern recognition. This pattern of good correlation coupled with large errors is characteristic of PCP, as the correlation reflects the consistency in the temporal ordering of rainy season peaks across locations, while the MAE and RMSE quantify the magnitude of the phase shift (in days) of those peaks.

**Fig. 14:**
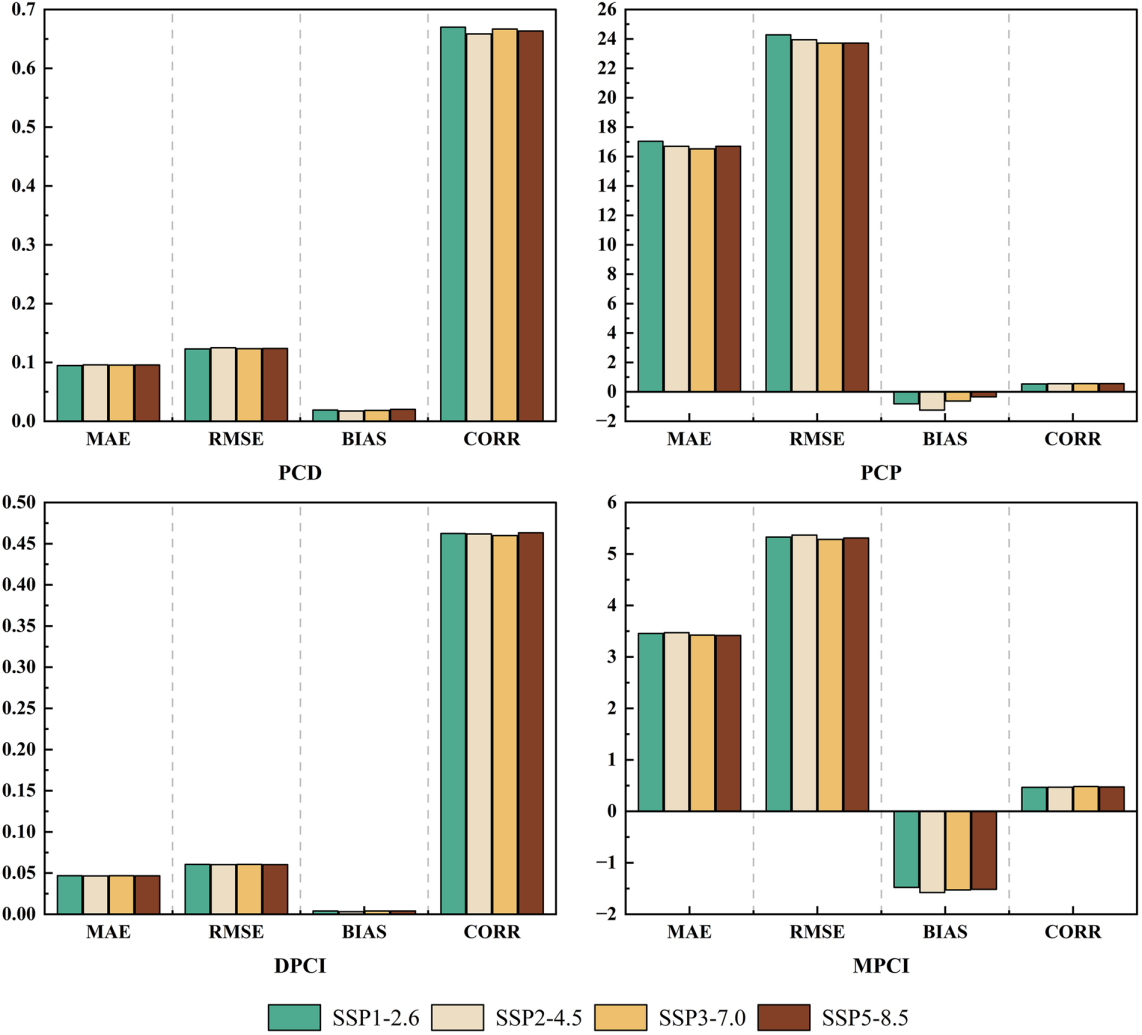
Evaluation of precipitation concentration indicators from downscaled CMIP6 data under SSP scenarios. The assessment compares the downscaled future data (2015–2020) against ground station observations over the same period. The sample size for each indicator and each SSP scenario is based on 651 stations over 6 years (2015–2020), resulting in 3,906 data points per indicator per scenario. Bar plots show the distribution of errors and correlation for each indicator across the four SSP scenarios.

Figure 14 evaluates performance variations of four precipitation concentration indicators in CMIP6 downscaled data across different SSP scenarios, using station observations as the reference baseline. PCD showed consistently low errors, with MAE ranging from 0.094 to 0.096 and RMSE from 0.122 to 0.125 across SSP scenarios, accompanied by minimal inter-scenario MAE variation. Systematic BIAS values ranged from 0.017 to 0.020, accompanied by moderate CORR values between 0.658 and 0.670. PCP exhibited pronounced spatiotemporal decoupling, characterized by the largest temporal errors (MAE: 16.517–17.037; RMSE: 23.708-24.277). Medium-low emission scenarios (SSP1-2.6 and SSP2-4.5) showed distinct negative BIAS (−1.237 to −0.625), indicating systematic underestimation of rainy season peak timing. CORR values increased gradually across scenarios (0.534–0.556), peaking under SSP5-8.5 (0.556). This pattern, consistent with the historical evaluation, indicates that the downscaled data capture the geographical seasonality well but exhibit a systematic phase shift in the timing of the precipitation concentration period. DPCI and MPCI exhibited contrasting performance patterns. Specifically, DPCI maintained robust error control (MAE: 0.046–0.047; RMSE: 0.060–0.061) but low CORR (0.459–0.463), underscoring persistent stochastic uncertainty in daily precipitation modeling. MPCI errors showed limited emission-driven variation (MAE: 3.416–3.471; RMSE: 5.283–5.366), while systematic negative BIAS (−1.577 to −1.477) highlighted limitations in capturing monthly cumulative extremes under high radiative forcing.

The initial evaluation in this section focused on temporal metrics to reflect the agreement between bias-corrected model outputs and observations. However, such a perspective is insufficient to fully demonstrate the reliability of model data for future spatial projections. To address this, we supplemented additional evaluation results by comparing the spatial patterns of precipitation concentration indicators between bias-corrected models and observations, combined with statistical significance analysis.

**Fig. 15:**
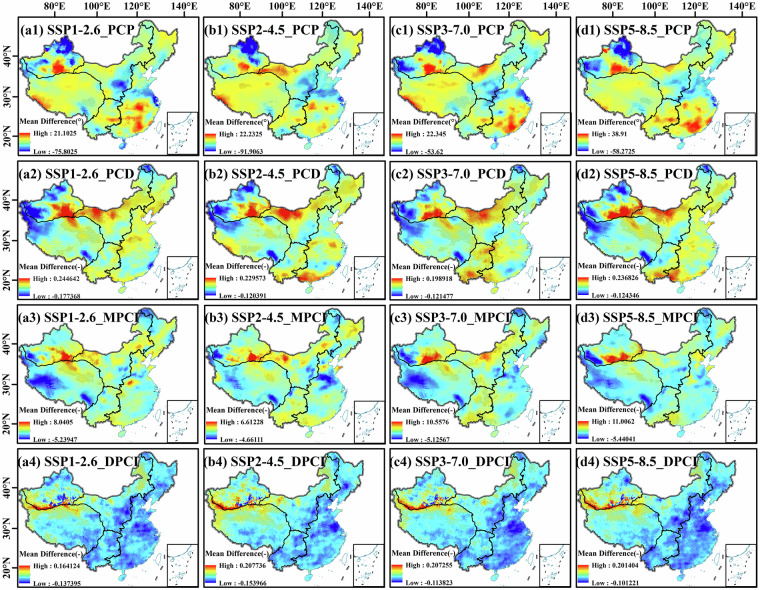
Spatial mean differences of precipitation concentration indicators between bias-corrected GCMs (under SSP1-2.6, SSP2-4.5, SSP3-7.0, and SSP5-8.5 scenarios) and CN05.1 observations during 2015–2022. The color gradient denotes the mean difference (model minus observation): blue indicates the model underestimates the indicator, while red indicates the model overestimates the indicator. Semi-transparent black triangles mark grid points where the difference is statistically significant at the 90% confidence level (two-sample *t*-test, *p* < 0.1).

As shown in Fig. 15, by comparing the spatial average differences of the precipitation concentration indices between the bias-corrected MME (under SSP1-2.6, SSP2-4.5, SSP3-7.0, and SSP5-8.5 scenarios) and the CN05.1 observation data from 2015 to 2022, we can further analyze the spatial heterogeneity of the index and the model’s ability to characterize observational features. For the PCP index, the distribution of differences between the bias-corrected model and observations presents a spatial pattern of “the precipitation concentration period is later in the southeast and earlier in the northwest”. Specifically, the southeast region is mostly red (model overestimation, delayed precipitation concentration period), and the northwest region is mostly blue (model underestimation, advanced precipitation concentration period). The semi-transparent black triangles (p < 0.1) in areas such as northern XJ and the central part of the EP indicate that these differences are statistically significant. The PCD index shows a spatial pattern of “overestimation in ILP, southern XJ, and southern coastal regions, and underestimation in western XJ and parts of western TP”. The distribution of significant triangles indicates that the differences between the model and observations are statistically significant, and the corrected model has statistical reliability in simulating the characteristics of PCD that “concentration is high in the north and low in the west”. The spatial pattern of the MPCI index of “overestimation in ILP and southern XJ, and underestimation in southwestern TP” is consistent across different SSPs. The dense distribution of significant triangles indicates that the corrected model has reference value in characterizing the feature of MPCI that “the comprehensive precipitation concentration index is relatively high in ILP and southern XJ”. The DPCI index shows an overall underestimation trend, with overestimation at the junction of XJ and TP. The coverage of significant triangles in the corresponding areas reflects that the model has statistical reliability in characterizing the regional differentiation law of DPCI.

## Usage Notes

To facilitate automated processing, users should note the following technical specifications: the temporal coverage spans 1961–2022 (historical) and 2015–2100 (future); missing values are encoded as NaN; the spatial reference is the WGS-84 datum; and the temporal basis is the standard Gregorian calendar.

### Data application scope

MPCID is designed for the study of precipitation concentration across mainland China over the period 1961 to 2100. It enables detailed analysis of spatiotemporal patterns in precipitation concentration, allowing researchers to characterize how precipitation aggregates across regions and seasons. Specifically, it can be applied to investigate climate change impacts on precipitation concentration trends in both historical and future periods, providing critical insights for assessing the effects of extreme precipitation events on hydrological regimes and agricultural systems.

### Advantages of the dataset

MPCID possesses several notable advantages. First, it integrates historical station and grid observations (1961 to 2022) with CMIP6-based future projections (2015 to 2100) across four SSP scenarios, bridging the historical-future research gap and overcoming limitations of traditional approaches that rely on fragmented station data. Second, statistical downscaling frameworks generate high-resolution (0.25°, ~30 km) projections, thereby enhancing spatial reliability for regional climate analyses. Third, it includes multi-dimensional precipitation concentration indicators, which collectively quantify spatiotemporal precipitation patterns and support comprehensive climate risk assessments.

### Indicator features and considerations

Users are advised to consider the unique limitations and characteristics of each indicator when using the dataset. DPCI exhibits moderate error control but limited daily-scale correlation, indicating limitations in capturing short-term precipitation variability. MPCI exhibits reduced sensitivity to extreme precipitation events, characterized by substantial errors and pronounced negative bias in monthly-scale analyses. PCP maintains high temporal correlation but exhibits large spatiotemporal discrepancies in rainy season peak identification, primarily resulting from temporal misalignment in peak detection.

### Considerations for spatial interpretation

Users should be aware that the gridding process, which employs KD-Tree with IDW for station-based interpolation and bilinear resampling for GCMs outputs, may smooth local extremes and underestimate precipitation gradients in areas of complex terrain or where meteorological stations are sparse. We strongly advise users to interpret local patterns and extreme values of precipitation concentration indicators in conjunction with the underlying station density and topographic features.

### Limitations

Sparse observational networks in remote or high-altitude regions constrain the accuracy of model calibration, thereby introducing uncertainties in the projected trends of precipitation concentration indicators. Statistical downscaling methods, which rely on CMIP6 model outputs, inherit systematic biases in simulating extreme precipitation events. Additionally, the dataset’s dependence on scenario-specific emission assumptions amplifies uncertainties, as socioeconomic trajectories and policy interventions under SSPs are inherently challenging to predict with precision. Furthermore, as identified in the technical validation, the statistical downscaling processes, particularly the CI-integrated ones, tend to produce a positive bias in the upper tail of the precipitation distribution. Users focusing on extreme precipitation events and their concentration are advised to interpret these results with caution and to consider the quantile-dependent biases discussed in Section downscaling and evaluation of future projected data.

### Research significance and future directions

Despite these limitations, the dataset serves as a valuable cross-disciplinary resource. It establishes a foundational framework for meteorological and geographical research, facilitating in-depth exploration of precipitation-environment system interactions. Future studies can build upon this dataset to integrate high-resolution regional climate models with multi-source observational data, thereby refining the simulation of precipitation concentration indicators. Researchers may also develop coupled analytical frameworks that link precipitation dynamics to socioeconomic drivers, enabling comprehensive assessments of multidimensional impacts on agriculture, ecosystems, and urban planning. Additionally, efforts can focus on establishing robust extreme precipitation prediction systems and formulating targeted climate resilience strategies to address evolving environmental challenges.

To enhance the long-term scientific value and adaptability of MPCID, we have designed a dynamic update and recalibration framework based on extended observational datasets. For the updated observational data source, we will adopt the CMFD V2.0 (China Meteorological Forcing Data Version 2.0, 10.11888/Atmos.tpdc.302088) as the core observational support for future iterations^44^. This dataset covers 1951–2024 with a 0.1° spatial resolution and 3-hourly temporal resolution, providing more comprehensive precipitation and auxiliary meteorological variables than the current CN05.1 dataset (1961–2022, 0.25°). The extended historical period (1951–1999) will improve the statistical reliability of CDF fitting in the QDM method, while the latest 2023–2024 data will extend the baseline assessment window for bias-corrected projections. For the periodic update mechanism, we plan to recalibrate the multi-SSP scenario projections every 2–3 years. Each update will involve first reconstructing the historical CDF using the latest observational data; second re-optimizing QDM parameters to preserve climate change signals while eliminating new GCMs biases; third validating the recalibrated data against extended baseline observations; fourth releasing updated versions with detailed version logs. For cross-validation with CN05.1 updates, we will monitor updates to the CN05.1 dataset and cross-validate it with CMFD V2.0 to ensure consistency of historical baselines, further reducing uncertainties in recalibration.

This framework ensures that MPCID remains aligned with the latest observational evidence, continuously improving the accuracy of future precipitation concentration projections for hydrological, agricultural, and climate adaptation research.

## Data Availability

The dataset, including detailed methodological descriptions and additional validation results, are available with the main paper.

## Code availability

Custom code used for statistical downscaling and precipitation concentration indicator calculation are deposited in a permanent repository. The repository is Zenodo, and the DOI for this Zenodo repository is 10.5281/zenodo.17503458. The code is released under the Creative Commons Attribution 4.0 International License (CC BY 4.0). This license permits unrestricted reuse of the code, including commercial use, on the condition that appropriate attribution is provided.
